# Crosstalk of MAP3K1 and EGFR signaling mediates gene-environment interactions that block developmental tissue closure

**DOI:** 10.1016/j.jbc.2024.107486

**Published:** 2024-06-18

**Authors:** Jingjing Wang, Bo Xiao, Eiki Kimura, Maureen Mongan, Wei-Wen Hsu, Mario Medvedovic, Alvaro Puga, Ying Xia

**Affiliations:** Department of Environmental and Public Health Sciences, University of Cincinnati, College of Medicine, Cincinnati, Ohio, USA

**Keywords:** AHR, developmental tissue closure, dioxin, EGFR, epithelial morphogenesis, gene-environment interactions, the S1PR-MAP3K1-JNK pathway

## Abstract

Aberrant regulation of signal transduction pathways can adversely derail biological processes for tissue development. One such process is the embryonic eyelid closure that is dependent on the mitogen-activated protein kinase kinase kinase 1 (MAP3K1). *Map3k1* KO in mice results in defective eyelid closure and an autosomal recessive eye-open at birth phenotype. We have shown that *in utero* exposure to dioxin, a persistent environmental toxicant, induces the same eye defect in *Map3k1*^+/−^ heterozygous but not WT pups. Here, we explore the mechanisms of the *Map3k1* (gene) and dioxin (environment) interactions (GxE) underlying defective eyelid closure. We show that, acting through the aryl hydrocarbon receptor, dioxin activates epidermal growth factor receptor signaling, which in turn depresses MAP3K1-dependent Jun N-terminal kinase (JNK) activity. The dioxin-mediated JNK repression is moderate but is exacerbated by *Map3k1* heterozygosity. Therefore, dioxin exposed *Map3k1*^*+/−*^ embryonic eyelids have a marked reduction of JNK activity, accelerated differentiation and impeded polarization in the epithelial cells. Knocking out *Ahr* or *Egfr* in eyelid epithelium attenuates the open-eye defects in dioxin-treated *Map3k1*^*+/−*^ pups, whereas knockout of *Jnk1* and *S1pr* that encodes the sphigosin-1-phosphate (S1P) receptors upstream of the MAP3K1-JNK pathway potentiates the dioxin toxicity. Our novel findings show that the crosstalk of aryl hydrocarbon receptor, epidermal growth factor receptor, and S1P-MAP3K1-JNK pathways determines the outcome of dioxin exposure. Thus, gene mutations targeting these pathways are potential risk factors for the toxicity of environmental chemicals.

The formation of a complex multicellular organism is one of the most fascinating processes in biology. These processes require signal transduction pathways to effectively transmit developmental cues to regulate cellular activities at the correct time and space. The mitogen-activated protein kinase (MAPK) pathway is an evolutionarily conserved signaling mechanism that exerts global control of developmental processes (1). The pathway consists of a three-tiered kinase module, including a MAPKK kinase (MAP3K), a MAPK kinase (MAP2K) and a MAPK. For each module, the endogenous and environmental signals activate the MAP3Ks that in turn phosphorylate the MAP2Ks to further phosphorylate and activate the MAPKs. The MAP3K is a family of 20 structurally related serine-threonine protein kinases, playing a crucial role in determining specificity of MAPK activation (2). In this capacity, each MAP3K is responsive to a distinctive set of upstream signals and in turn activates the specific MAP2K-MAPK modules (3). Consistent with their specificity in signal transduction, the MAP3Ks exhibit highly cell type-specific and tissue-specific roles in development (4). The MAP3K1, for example, is dispensable for embryonic survival, but uniquely needed for embryonic eyelid closure (5).

Mouse eyelid development starts at embryonic day 11.5 (E11.5), where the periocular epithelium derived from the surface ectoderm folds at the junction of the future conjunctiva and cornea, forming the eyelid buds. The eyelid buds elongate as embryo grows, and the epithelia at the eyelid leading edge extend centripetally. By E15.5, the opposing eyelid epithelium makes contact and fuses with each other, forming an enclosed eyelid that covers the ocular surface (6, 7). The mouse eyelids remain fused at birth, and the upper and lower eyelids separate, resulting in eyes open at postnatal day 12 to 14, a time point coinciding with developmental maturation of the major ocular surface tissues. Thus, mice are normally born with eyes closed; however, the newborn pups display an “eye-open” at birth (EOB) phenotype when the eyelids fail to fuse prenatally (8). Because the failure of eyelid closure does not impair prenatal survival and the EOB phenotype is distinct and easily detectable (9), mouse strains with eyelid closure defects are convenient research tools to elucidate the molecular mechanisms of developmental tissue closure (10, 11, 12, 13, 14).

Compelling genetic data have shown that *Map3k1* loss-of-function mutation is autosomal recessive for the EOB phenotype (15, 16, 17, 18). While the heterozygous mice have normal eyelid development, the homozygous KOs exhibit an EOB defect with 100% penetrance. The same EOB defects are found in mice lacking MAP3K1 kinase domain, suggesting that the kinase activity in MAPK signaling is essential for eyelid closure. In alignment with this suggestion, MAP3K1 is found required for optimal activation of the Jun N-terminal kinases (JNKs) in epithelial cells at the leading edge of the embryonic eyelids (16, 19). Genetic testing lends further support to the existence of a MAP3K1-JNK pathway in eyelid morphogenesis. While neither the *Map3k1* heterozygosity (*Map3k1*^*+/−*^) nor the *Jnk1* KO (*Jnk*^*−/−*^) and the *Jnk1*^*+/−*^*Jnk2*^*+/−*^ double heterozygous mice exhibit the EOB phenotype, the *Map3k1*^*+/−*^*Jnk1*^*−/−*^ and *Map3k1*^*+/−*^*Jnk1*^*+/−*^*Jnk2*^*+/−*^ compound mutants have the defects (20). The genetic nonallelic noncomplementation strongly supports that *Map3k1*, *Jnk1*, and *Jnk2* gene products function in the same molecular pathway in the regulation of embryonic eyelid closure (21).

2,3,7,8-tetrachlorodibenzo-para-dioxin (TCDD) is an organochlorinated pollutant and the prototype of dozens of ubiquitous environmental compounds, known collectively as dioxin-like chemicals. These chemicals are biopersistent and ubiquitous in the environment; thus, human exposure is inevitable. Exposure of dioxin-like chemical has been linked to irregularities of many crucial life functions, including damage to the immune system, cardiovascular disease, diabetes, hormone dysfunction, and cancer (22). The biological effects of TCDD are mediated by the aromatic hydrocarbon receptor (AHR), a ligand activated basic-region helix–loop–helix-per-ARNT-sim transcription factor (23, 24). After binding with TCDD, the AHR translocates from the cytoplasm to the nucleus where it heterodimerizes with the AHR nuclear translocator and/or interacts with other transcription factors to, directly or indirectly, regulate gene expression (25, 26, 27, 28). AHR-regulated genes are responsible for most of the developmental toxicities of TCDD (24, 29, 30, 31, 32, 33); however, there is evidence suggesting that genes outside of the *Ahr* gene battery can modify the toxicity, resulting in species and individual variations, and cell type-specific responses (34, 35). The genetic modification mechanism is a trending topic of investigation to understand the complexity and individual diversity associated with TCDD toxicity.

When studying the developmental toxicity of TCDD in mice, we came across an observation that its exposure *in utero* induced an open-eye defect in heterozygous *Map3k1*^*+/−*^ pups. By contrast, exposure did not cause the defect in WT pups and in pups carrying *Dkk2* loss-of-function mutations that target the wingless-related integration site signaling pathways (36). In the current work, we investigated the mechanisms of the specific interactions between gene (*Map3k1*) x environment (TCDD) (GxE) in tissue closure abnormalities. We found that the toxicity of TCDD was mediated through the AHR to activate the epidermal growth factor receptor (EGFR)—extracellular signal regulated kinase (ERK) pathway. This in turn led to a modest JNK inhibition that was insufficient to cause an EOB phenotype. *Map3k1* heterozygosity also decreased JNK activity, and it acted additively or synergistically with TCDD to further inactive JNK, resulting in eyelid closure defects in TCDD-exposed *Map3k1*^*+/−*^ pups. We additionally identified the sphingosine 1-phosphate (S1P) receptors as upstream of the MAP3K1-JNK pathway and that knockout of *Jnk1*-or *S1pr2/3* also sensitized the embryonic eyelids to TCDD-induced closure defects. Thus, the gene (*Map3k1* heterozygosity) and the environment (TCDD) converge to inhibit JNK signaling through intertwining crosstalk of multiple signaling pathways.

## Results

### Ocular surface epithelial AHR mediates TCDD toxicity

**Figure 1:**
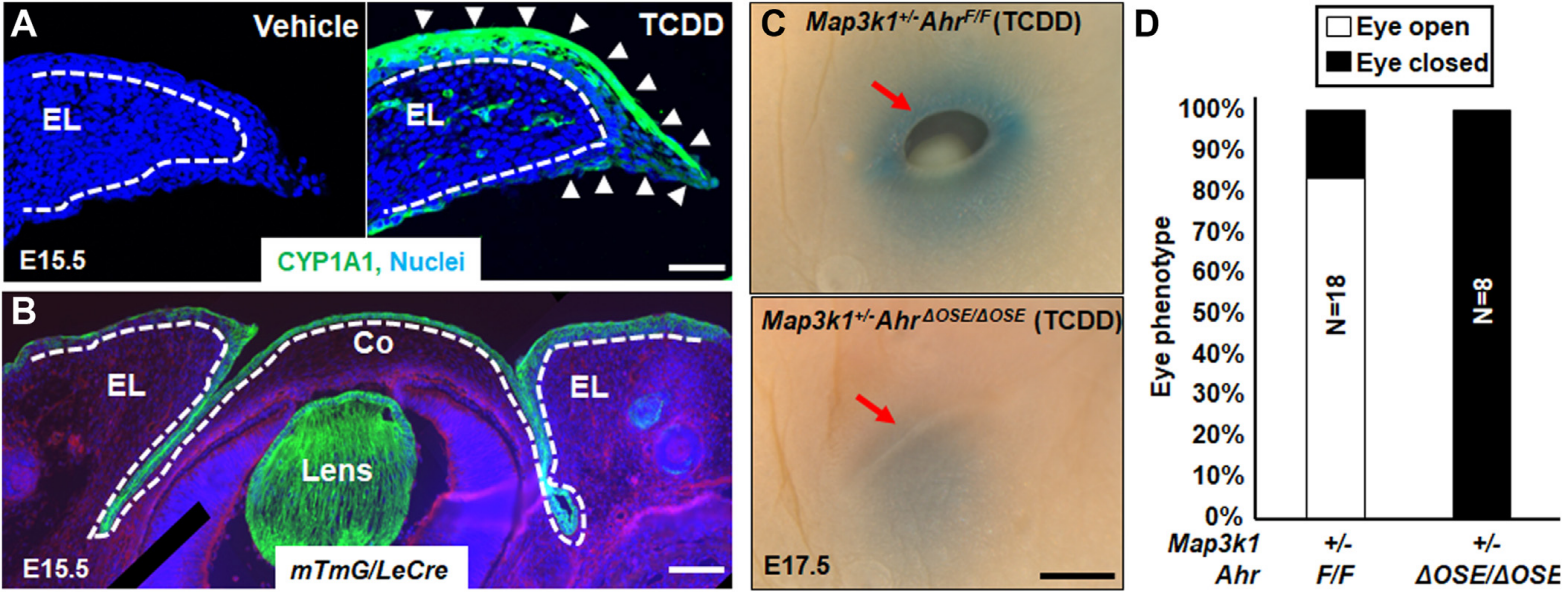
**Eyelid epithelial AHR is required for TCDD toxicity in embryonic eyelid closure.***A*, immunofluorescent staining using anti-CYP1A1 (*green*) and Hoechst (*blue*) for nucleus of the eyelids in WT E15.5 embryos with or without TCDD exposure (50 μg/kg body weight) at 11.5 days. *Dash lines* mark basement membrane that separated the eyelid epithelium and the underneath dermis; *white arrows* point at CYP1A1 positive staining. *B*, Hoechst (*blue*) staining for nucleus of eyes of the E15.5 *mTmG*/*Le-Cre* embryos. Cre expressing, *i.e.*, GFP positive, cells were located at the ocular surface epithelium (OSE), but not stroma of eyelid (EL), cornea (Co), and lens. The *dotted lines* mark the basement membrane that separated the epithelium and the stroma. Pups exposed at E11.5 to TCDD (250 μg/kg body weight) were collected at E17.5, and their eyes (*C*) were photographed, *red arrow*, eyelid leading edge, and (*D*) quantification of eyes displaying open eye phenotype in *Map3k1*^*+/−*^*Ahr*^*F/F*^ (N = 18) and *Map3k1*^*+/−*^*Ahr*^*ΔOSE/ΔOSE*^ (N = 8) fetuses. N= number of pups of the indicated genotype. The scale bars represent 50 μm in *A*, 100 μm in *B*, and 500 μm in *C*. AHR, aryl hydrocarbon receptor; MAP3K1, mitogen-activated protein kinase kinase kinase 1; OSE, ocular surface epithelium; TCDD, 2,3,7,8-tetrachlorodibenzo-para-dioxin.

Because AHR functions in a highly cell type- and tissue-specific manner (37, 38, 39), we wanted to first determine if it was responsive to TCDD in the embryonic eyelids. For this objective, we assessed the expression of the prominent AHR target gene *Cyp1a1*, encoding Cytochrome P450 1a1. Most abundant CYP1A1 expression was found in the suprabasal epithelia on the exterior side of the eyelids in E15.5 embryos collected from pregnant dams treated with TCDD (50 μg/kg body weight) on E11.5. The expression was also detectable, albeit less abundantly, in the inferior epithelia and the stroma of the exposed embryonic eyelids (Fig. 1*A*). As expected, CYP1A1 expression was undetectable in the eyelids of vehicle-treated embryos.

To address the role of the epithelial AHR in TCDD toxicity, we knocked out the *Ahr* gene in the developing ocular surface epithelium (OSE) by crossing *Ahr*^*F/F*^ with *Le-Cre* mice. The *Le-Cre* transgene drives Cre recombinase expression in the developing OSE (40). When tested in the *mTmG* reporter embryos, *Le-Cre* mediated GFP expression specifically in the OSE, *i.e.* epithelium, but not the stroma, of the eyelid, cornea, and lens (Fig. 1*B*). The *Ahr*^*F/F*^*Le-Cre*, *i.e.*, OSE-specific *Ahr* KO (*Ahr*^*ΔOSE*^), were subsequently used to make *Map3k1*^*+/−*^*Ahr*^*F/F*^ and *Map3k1*^*+/−*^*Ahr*^*ΔOSE*^ compound mutants, both of which had normal eyelid closure in the absence of TCDD exposure. When dams were treated with high-dose (250 μg/kg) TCDD, 15 out of 18 (84%) *Map3k1*^*+/−*^*Ahr*^*F/F*^ E17.5 fetuses exhibited the open-eye phenotype, whereas none of *Map3k1*^*+/−*^*Ahr*^*ΔOSE/ΔOSE*^ fetuses (N = 8) had the phenotype (Fig. 1, *C* and *D*).

Another piece of evidence suggesting AHR-mediated TCDD toxicity was the observations that all *Map3k1*^*+/−*^ pups on a congenic C57BL/6 (B6) background developed the eyelid closure defects when exposed to 50 μg/kg TCDD, but the *Map3k1*^*+/−*^*Ahr*^*F/F*^ pups showed the defects only when treated with 200 μg/kg or higher doses (Fig. S1). The differences in TCDD susceptibility are likely the result of strain specific *Ahr* gene polymorphisms that give rise to AHR proteins with different ligand binding affinities (41). The *Ahr* gene in B6 strain carries *Ahr*^*b1*^ alleles that encode a high-affinity receptor, whereas the *Ahr* locus in *Ahr*^*F*^ mice is derived from 129/SvJ strain that carries *Ahr*^*d*^ alleles encoding a low-affinity receptor (42). Thus, the ligand binding affinity of AHR determines sensitivity of the eyelids to TCDD toxicity.

### Activation of the TCDD-AHR axis induces EGFR signaling

As the TCDD-AHR axis exerts biological effects through transcriptional regulation of gene expression (43), we set out to determine TCDD-AHR-dependent transcriptome in HaCaT, an aneuploid immortal human epithelial cell line (44). These cells possess a functional TCDD-AHR signaling, because TCDD induces a marked *CYP1A1* mRNA that is abolished by treatment with CH223191, an AHR antagonist (Fig. S2*A*). RNA-seq of the HaCaT cells treated with TCDD and CH22391 led to the identification of a total of 3600 genes differentially expressed in a manner dependent on the TCDD-AHR pathway. Among them, 455 genes were upregulated or downregulated by 2-fold or above (Fig. S2, *B* and *C*). Ingenuity pathway analyses showed that cell cycle regulation and xenobiotic AHR signaling were the top TCDD-AHR upregulated functions, consistent with that reported previously (38, 45) (Fig. S2*D*). On the other hand, epithelial-to-mesenchymal transition in development was identified as a top TCDD-AHR repressed function.

**Figure 2:**
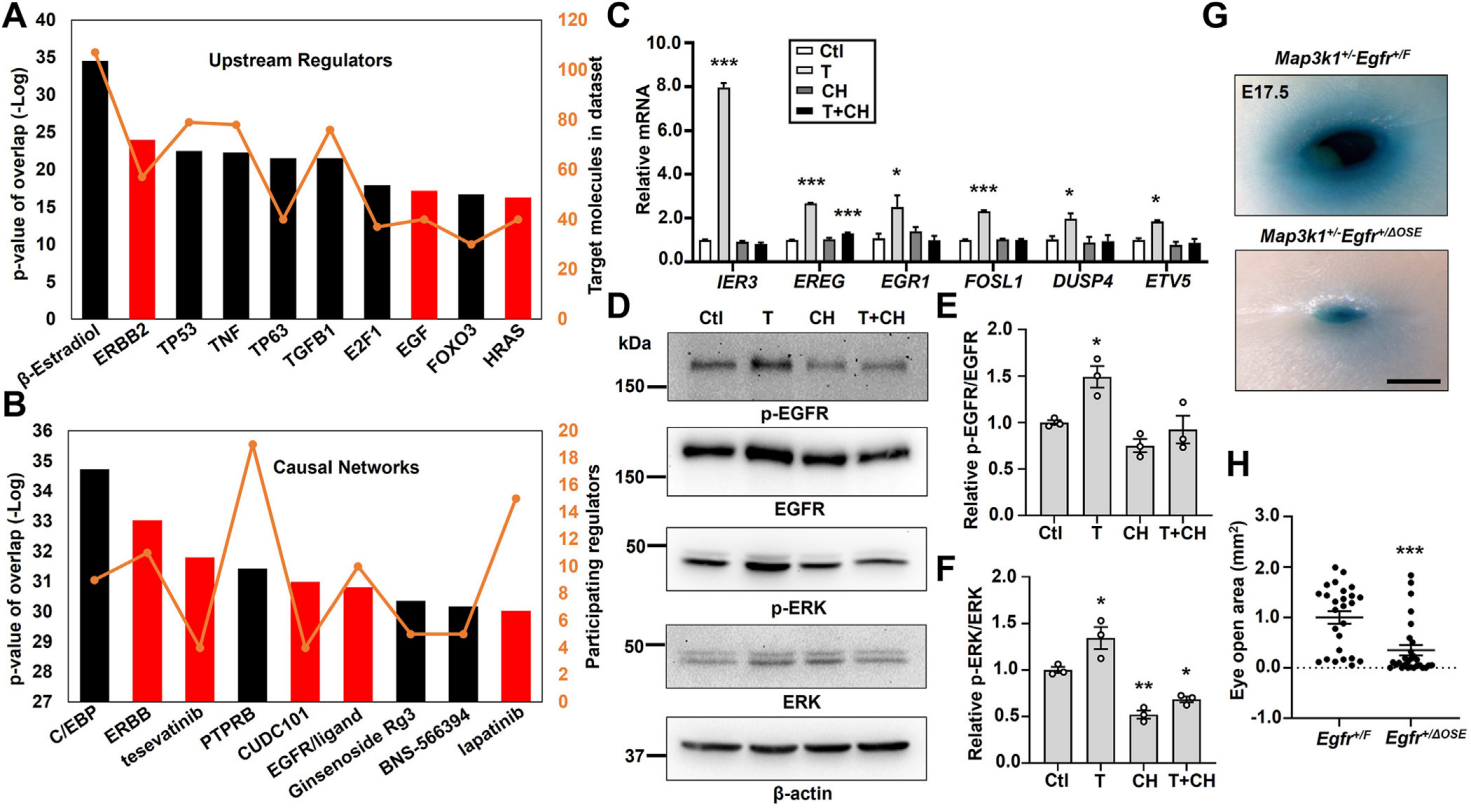
**The TCDD-AHR axis activates EGFR signaling.** The TCDD-AHR axis upregulated genes were subjected to ingenuity pathway analyses to identify (*A*) top upstream regulators and (*B*) top causal networks, with relative *p*-value (*bars*) and number of participating molecules (*orange line*). The EGFR signaling relevant upstream regulators and causal networks are labeled as *red bars*. *C*, selective gene expression was measured by qRT-PCR using RNA isolated from HaCaT cells treated with vehicle (DMSO, Ctl), TCDD (T, 10 nM), CH223191 (CH, 10 μM), and TCDD plus CH223191 (T+CH). Relative expression was calculated using the housekeeping gene *GAPDH* as an internal control and compared to that in Ctl set as 1. *D*, Western blot analyses of the EGFR pathway activity using antibodies for phosphor (p) and total EGFR and ERK, and β-actin as a loading control. Quantification of (*E*) p-EGFR/EGFR and (*F*) p-ERK/ERK ratio of the immunoblotting data, with ratio in Ctl set as 1. The TCDD-exposed *Map3k1*^*+/−*^*Egfr*^*+/F*^ (N = 13) and *Map3k1*^*+/−*^*Egfr*^*+/ΔOSE*^ (N = 14) fetuses were collected at E17.5 and their eyes were (*G*) photographed and representative images were shown. The scale bar represents 500 μm, and (*H*) the areas of open eye were measured. Results were shown as mean ± SEM of at least three independent experiments (N ≥ 3). ∗*p* < 0.05, ∗∗*p* < 0.01 and ∗∗∗*p* < 0.001 were considered statistically significant compared to Ctl (in *C*, *E*, and *F*) and TCDD-treated *Map3k1*^*+/−*^*Egfr*^*+/F*^ group (in *H*). AHR, aryl hydrocarbon receptor; DMSO, dimethyl sulfoxide; EGFR, epidermal growth factor receptor; ERK, extracellular signal regulated kinase; MAP3K1, mitogen-activated protein kinase kinase kinase 1; OSE, ocular surface epithelium; qRT-PCR, quantitative reverse transcription PCR; TCDD, 2,3,7,8-tetrachlorodibenzo-para-dioxin.

Further analyses of the transcriptome using the Upstream Regulator and Causal Network, which are designed to elucidate the biological causes and probable downstream effects (46), led us to identify EGFR signaling as being altered by the TCDD-AHR pathway. The Upstream Regulator Analyses enables prediction of the cascades and transcription factors to explain the gene expression signatures in the datasets. The Causal Network extends the analyses to include regulators that are indirectly connected to targets in the datasets. These advanced analyses identified top TCDD-AHR affected molecules/pathways, several of which are implicated in EGFR signaling (Fig. 2, *A* and *B*). Specifically, TCDD induced the expression of EGFR upstream regulators, such as ERBB2, EGF, and HRAS, and perturbed EGFR gene networks, including ERBB, tesevatinib, CUDC101, and lapatinib (Fig. 2, *A* and *B*). Some genes in these functional categories, such as *IER3, EREG, EGR1, FOSL1, DUSP4*, and *ETV5*, were validated for their expression being induced by TCDD and the induction was abolished by the AHR antagonist (Fig. 2*C*).

To verify the effect of TCDD on EGFR signaling, we measured EGFR tyrosine 1173 phosphorylation, a hallmark of receptor activation (47). TCDD-treated HaCaT cells had a slight (1.5-fold) yet persistent increase of p-EGFR in comparison to vehicle-treated cells, and the increase was abolished by the AHR antagonist (Fig. 2, *D* and *E*). Similarly, the phosphorylation of ERK, a downstream activation target of EGFR (48), was induced by TCDD in a manner dependent on AHR (Fig. 2, *D* and *F*).

EGFR regulates epithelial cell proliferation that is required for embryonic eyelid closure and the *Egfr* homozygous KO, either in germline or in OSE, results in the EOB phenotype (49). To test genetically the roles of ocular surface EGFR in TCDD-*Map3k1* crosstalk, we made *Egfr*^*+/ΔOSE*^ mice, which had one *Egfr* allele deletion in the OSE cells but normal eyelid closure. We subsequently generated *Map3k1*^*+/−*^*Egfr*^*+/F*^ and *Map3k1*^*+/−*^*Egfr*^*+/ΔOSE*^ pups that were also normal in eyelid closure in the absence of TCDD exposure. After exposed to TCDD *in utero*, most of the EGFR WT (*Egfr*^*+/F*^) *Map3k1*^*+/−*^ fetuses developed the open-eye defects; however, the *Egfr*^*+/ΔOSE*^*Map3k1*^*+/−*^ fetuses, in which EGFR expression in OSE was presumably reduced by 50%, were less defective (Fig. 2*G*). The severity of the open-eye defect, *i.e.*, size of eye opening, varied among individuals, but was overall significantly milder and decreased by 64% in *Map3k1*^*+/−*^*Egfr*^*+/ΔOSE*^ compared to that in *Map3k1*^*+/−*^*Egfr*^*+/F*^ fetuses (Fig. 2*H*). Hence, TCDD acts at least partially through the epithelial EGFR to disrupt eyelid closure in *Map3k1*^*+/−*^ pups. In contrast, all *Map3k1* WT fetuses had normal eyelid closure in the presence or absence of *Egfr* heterozygosity and/or TCDD treatment.

### EGFR-ERK and MAP3K1-JNK form an inhibitory loop

**Figure 3:**
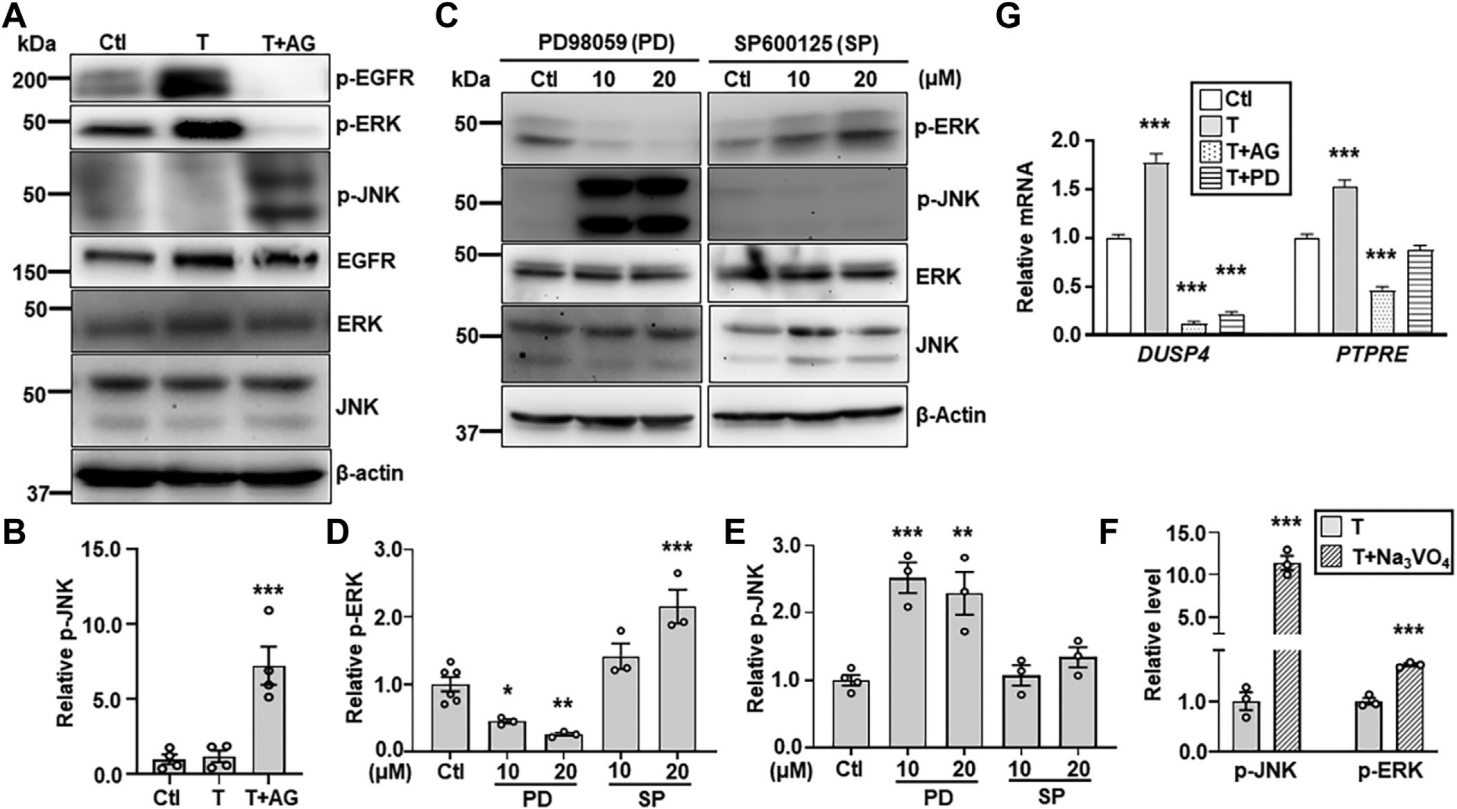
**The EGFR-ERK pathway inhibits JNK activities.** HaCaT cells treated with vehicle (DMSO, Ctl) and TCDD (T, 10 nM) in the presence or absence of the EGFR inhibitor, AG1478 (AG, 10 μM), were (*A*) subjected to immunoblotting for p-EGFR, p-ERK, p-JNK, total JNK, and β-actin, and (*B*) quantification for p-JNK using β-actin as a loading control. HaCaT cells treated with different concentrations of an ERK inhibitor, PD98059 (PD), and a JNK inhibitor, SP600125 (SP) were (*C*) examined by immunoblotting for p-ERK, p-JNK, and β-actin, and values of (*D*) p-ERK and (*E*) p-JNK were quantified and compared to that of β-actin. *F*, p-JNK, p-ERK, and β-actin were examined by immunoblotting in lysates of HaCaT cells treated with TCDD (T, 10 nM) in the presence and absence of phosphatase inhibitor Na_3_VO_4_ (0.5 mM). Values of relative p-JNK and p-ERK were quantified using β-actin. *G*, expression of *DUSP4* and *PTPRE* was examined by qRT-PCR in HaCaT cells treated with vehicle (DMSO, Ctl) and TCDD (T, 10 nM) in the presence and absence of 10 μM AG and PD. Relative mRNA of *DUSP4* and *PTPRE* was calculated using *GAPDH* as an internal control. Value of Ctl sample was set as 1. Data were shown as mean ± SEM of at least three independent experiments (N ≥ 3). ∗*p* < 0.05, ∗∗*p* < 0.01 and ∗∗∗*p* < 0.001 were considered significantly different compared to Ctl (in *B*, *D*, *E*, and *G*) and TCDD-treated cells (in *F*). DMSO, dimethyl sulfoxide; DUSP4, dual specificity phosphatase 4; EGFR, epidermal growth factor receptor; ERK, extracellular signal regulated kinase; JNK, Jun N-terminal kinase; PTPRE, protein tyrosine phosphatase epsilon; qRT-PCR, quantitative reverse transcription PCR; TCDD, 2,3,7,8-tetrachlorodibenzo-para-dioxin.

Treatment of HaCaT cells with AG1478, a specific EGFR inhibitor, abolished the basal and TCDD-induced phosphorylation of EGFR and ERK, but conversely, it markedly increased JNK phosphorylation (Fig. 3, *A* and *B*). Similarly, treatment of cells with PD98059, an ERK inhibitor, diminished the p-ERK, but induced the p-JNK (Fig. 3, *C* and *D*). While these observations suggested that the EGFR-ERK pathway led to the repression of JNK activity, we found that inhibition of JNK by SP600125 induced the p-ERK (Fig. 3, *C* and *E*), suggesting a mutual inhibitory relationship of JNK and ERK signaling.

When the HaCaT cells were treated with a nonspecific phosphatase inhibitor Na_3_VO_4_ (50, 51), there was a significant increase of p-JNK by 12-fold and p-ERK by 2-fold (Fig. 3*F*). Based on these observations, we asked whether TCDD induced protein phosphatases to inhibit JNK signaling (52). Searching the transcriptomics, we found that the TCDD-AHR pathway induced the expression of several phosphatase genes, including *DUSP4*, *ALPPL2*, *ALPP*, and *PTPRE* (Fig. S3, *A* and *B*). Among them, the dual specificity phosphatase 4 (DUSP4) and protein tyrosine phosphatase epsilon (PTPRE), are potent enzymes in dephosphorylation and inactivation of JNK (53, 54). Their expression was induced by TCDD in a manner also dependent on EGFR and ERK (Fig. 3*G*), suggesting that TCDD may act *via* EGFR-ERK to induce phosphatases for JNK repression.

**Figure 4:**
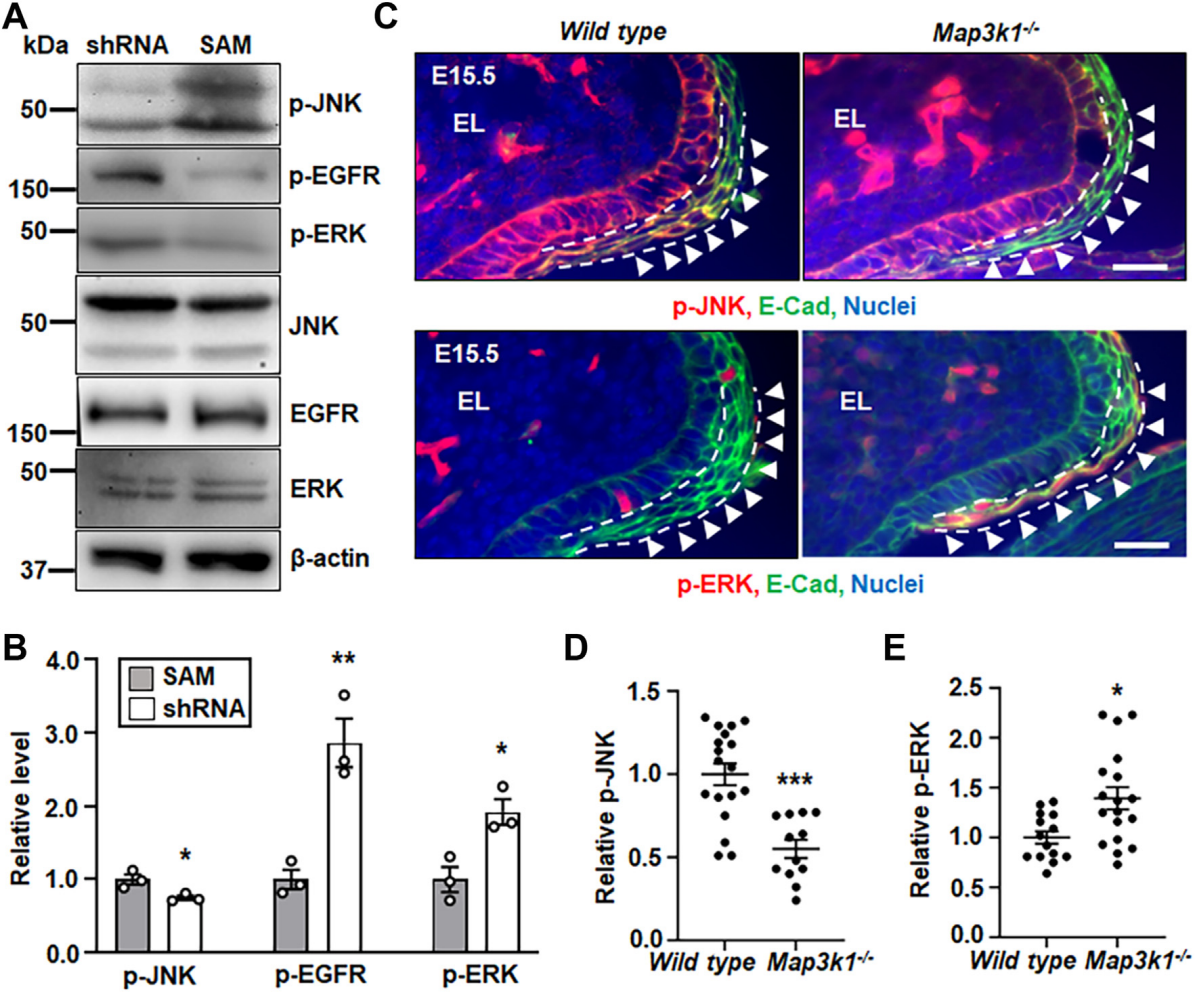
**The MAP3K1-JNK and EGFR-ERK pathways inhibit each other.** Lysates of the shRNA HaCaT and SAM HaCaT cells were examined by Western blotting for (*A*) p-JNK, p-EGFR, p-ERK, and β-actin, and (*B*) quantification of p-JNK, p-EGFR, and p-ERK using β-actin as a loading control. Levels in SAM HaCaT were set as 1. Data represented three independent experiments (N = 3) and were shown as mean ± SEM. *C*, immunofluorescence staining of WT and *Map3k1*^*−/−*^ E15.5 embryonic eyelids with anti-p-JNK (*red*, *top* panels) and anti-p-ERK (*red*, *bottom* panels), costained with anti-E-cadherin (*green*) that marks epithelial membrane, and Hoechst (*blue*) labels nuclei. Representative images were shown, the scale bars represent 50 μm. The (*D*) p-JNK, and (*E*) p-ERK, in the suprabasal epithelial cells, marked with *dash lines*, of the eyelid leading edge (*arrowheads*) were quantified and compared to that in WT, set as 1. At least three sections (N ≥ 3) per embryo and three embryos (N ≥ 3) of each genotype from different litters were examined. Data were shown as mean ± SEM. ∗*p* < 0.05, ∗∗*p* < 0.01 and ∗∗∗*p* < 0.001 were considered significantly different from SAM cells (in *B*) and WT embryos (in *D* and *E*). EGFR, epidermal growth factor receptor; ERK, extracellular signal regulated kinase; JNK, Jun N-terminal kinase; MAP3K1, mitogen-activated protein kinase kinase kinase 1; SAM, synergistic activation mediator.

As MAP3K1 is upstream of JNK signaling (55), we next assessed the involvement of MAP3K1 in the EGFR/ERK-JNK crosstalk. For an *in vitro* assessment, we made stable HaCaT cells in which MAP3K1 expression was either downregulated with a small hairpin RNA, *i.e.*, MAP3K1-deficient (shRNA), or specifically upregulated with the single guide RNA (sgRNA)/CRISPR/dCas9 synergistic activation mediator (SAM) system, *i.e.*, MAP3K1-competent (SAM) (Fig. S3*C*). In these cells, we showed that increased MAP3K1 expression in the SAM cells led to elevated p-JNK, whereas decreased MAP3K1 in shRNA cells was associated with higher levels of p-EGFR and p-ERK (Fig. 4, *A* and *B*). For the *in vivo* assessment, we examined the eyelids of WT and *Map3k1*-null E15.5 embryos. Compared to the WT embryos, the *Map3k1*^*−/−*^ embryos had reduced p-JNK but elevated p-ERK in the suprabasal epithelium near the inferior eyelid leading edge (Fig. 4*C*). Quantification of the immunostaining signals revealed a 45% reduction of p-JNK but 36% increase of p-ERK in *Map3k1*^*−/−*^ *versus* WT eyelids (Fig. 4, *D* and *E*). Together, the *in vitro* and *in vivo* data suggest a feed-forward inhibitory loop, in which EGFR-ERK represses MAP3K1-JNK, and the repression of MAP3K1-JNK potentiates EGFR and ERK activities that may in turn further inhibit JNK.

**Figure 5:**
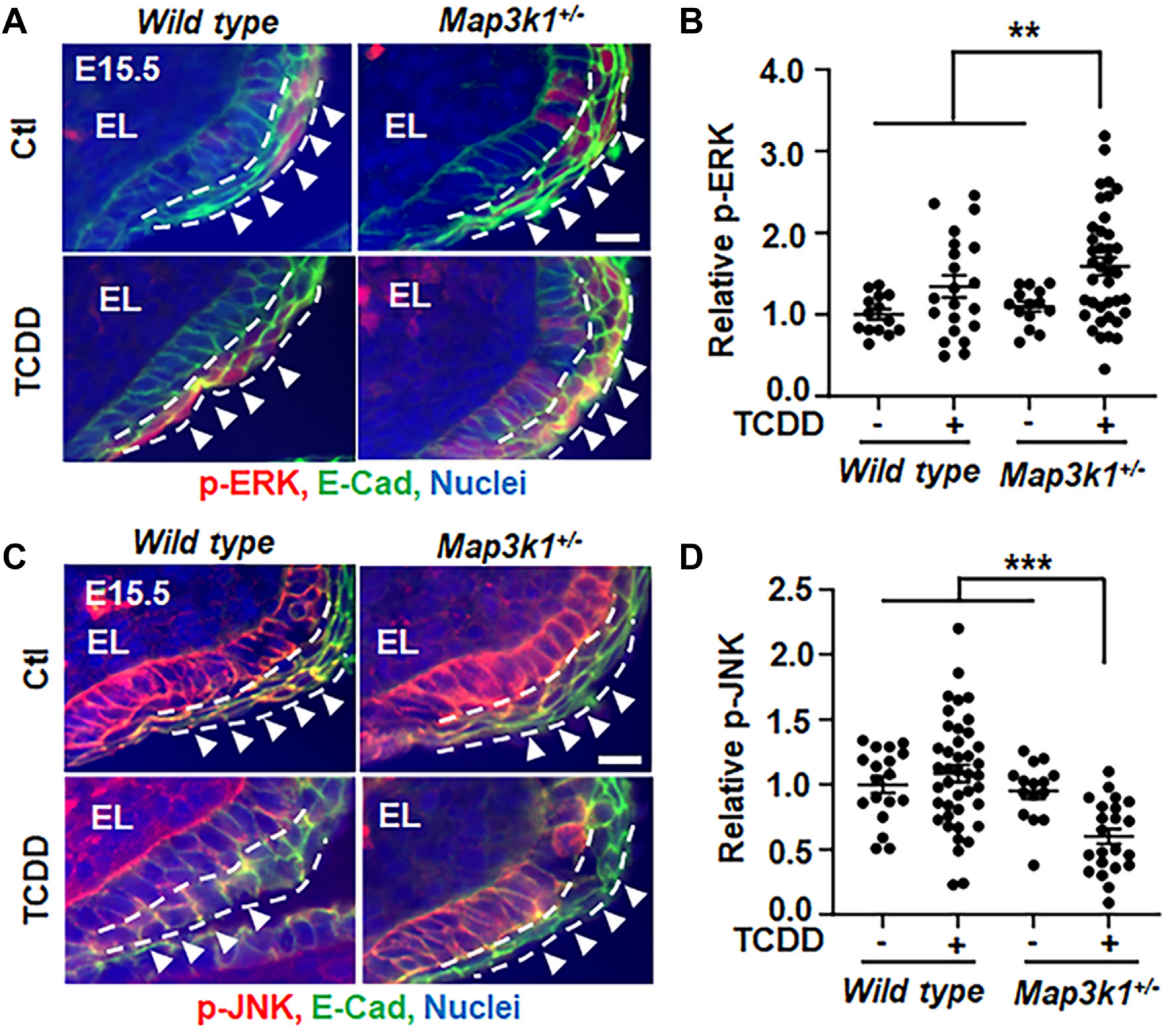
**TCDD plus *Map3k1* heterozygosity tilt the balance of JNK and ERK.** Eyelid tissues of WT and *Map3k1*^*+/−*^ E15.5 embryos with or without TCDD (50 μg/kg body weight) exposure were subjected to immunohistochemistry for (*A* and *B*) p-ERK (*red*) and (*C* and *D*) p-JNK (*red*). E-cadherin (*green*) and Hoechst (*blue*) were markers of epithelial membrane and nuclei, respectively. Representative images captured with a fluorescent microscopy were shown. The (*B*) p-ERK and (*D*) p-JNK in the suprabasal epithelium of the eyelid leading edge (*arrowheads*), marked with *dash lines*, were quantified. At least three sections (N ≥ 3) per embryo and three embryos (N ≥ 3) per genotype/treatment conditions were examined. Results were shown as mean ± SEM. ∗∗*p* < 0.01, ∗∗∗*p* < 0.001 represents significantly different from the unexposed groups and TCDD-treated WT group (in *B* and *D*). The scale bar represent 50 μm (*A* and *C*). ERK, extracellular signal regulated kinase; JNK, Jun N-terminal kinase; MAP3K1, mitogen-activated protein kinase kinase kinase 1; TCDD, 2,3,7,8-tetrachlorodibenzo-para-dioxin.

Through the feed-forward signaling loop, the small effects elicited by either TCDD or *Map3k1* heterozygosity might be substantially amplified when the two conditions are combined. This appeared to be the case as while the TCDD treatment (TCDD-treated WT) or the *Map3k1* heterozygosity (vehicle-treated *Map3k1*^*+/−*^) did not significantly change p-JNK and p-ERK from those in vehicle-treated WT, the TCDD-exposed *Map3k1*^*+/−*^eyelids had a marked increase of p-ERK and a significant decrease of p-JNK (Fig. 5, *A*–*D*).

### JNK deficiency is the molecular base of GxE interactions in tissue closure defects

**Figure 6:**
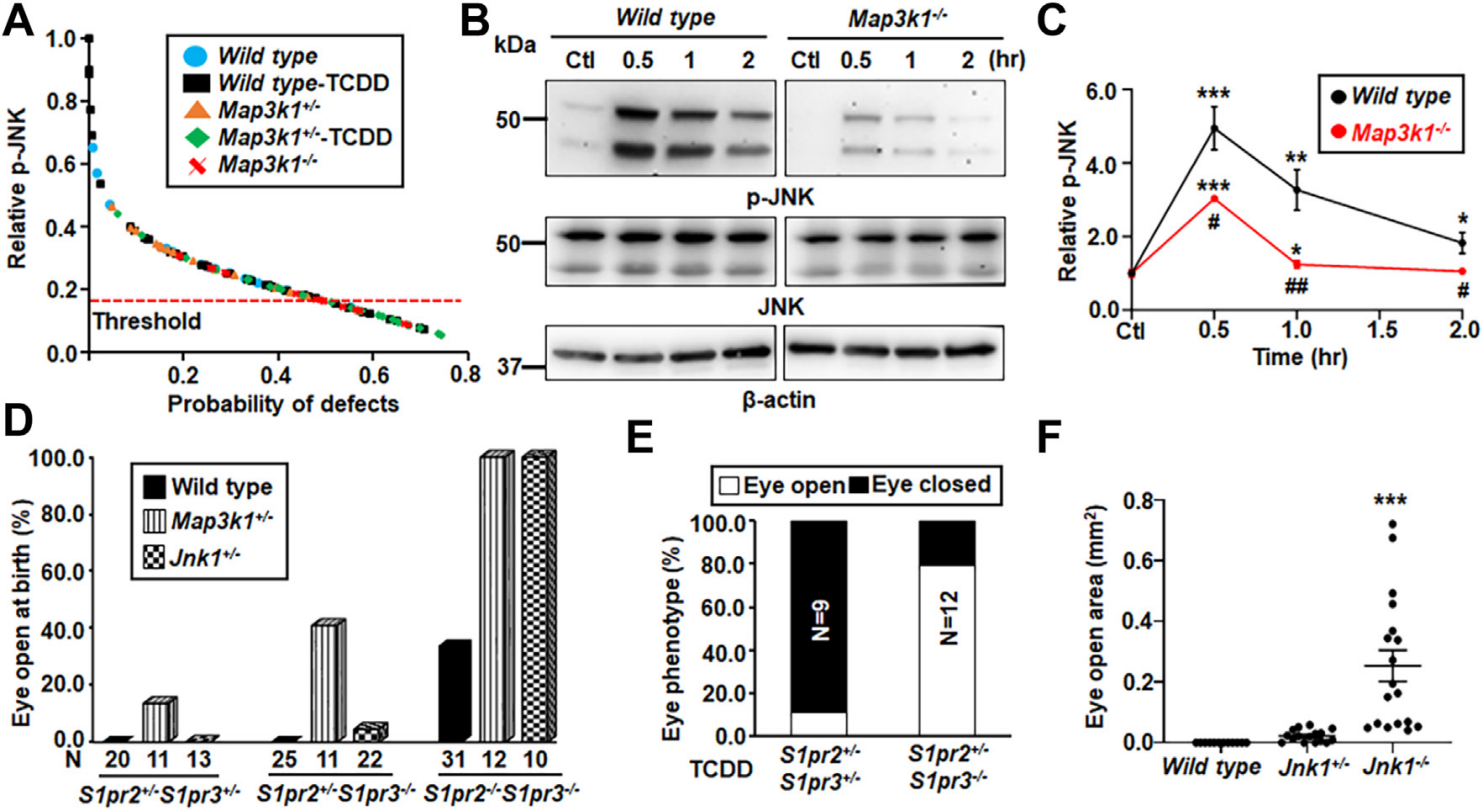
**Crosstalk of TCDD and the S1PR-MAP3K1-JNK1 pathway for embryonic eyelid closure.***A*, the levels of p-JNK in the eyelid epithelium were collected from E15.5 embryos of different genetic and exposure conditions as indicated. Data were analyzed by ImageJ software, and the relationship between the relative p-JNK level and the probability of the open-eye defects was calculated using a logistic regression model based on the binary outcome of eyelid closure, along with ROC analysis. Probability of defect was significant higher when p-JNK was below 0.17. *B*, The WT and *Map3k1*^*−/−*^ keratinocytes treated with vehicle (DMSO, Ctl) or 20 μM S1P for 0.5 to 2 h and p-JNK and β-actin were examined with Western blotting. *C*, quantification of p-JNK using β-actin as a loading control. Data are mean ± SEM of at least three independent experiments (N ≥ 3). The *S1pr2*-and *S1pr3*-compound mutant pups under *WT, Map3k1*^*+/−*^ and *Jnk1*^*+/−*^ genetic backgrounds as indicated (*D*) without and (*E*) with *in utero* exposure to 50 μg/kg TCDD on E11.5 were collected at E17.5. The pups were examined for the eyelid open/close. N = number of pups of the indicated genotype. *F*, the open eye areas were measured in E17.5 WT, *Jnk1*^*+/−*^ and *Jnk1*^*−/−*^ fetuses exposed to TCDD (50 μg/kg body weight) at E11.5. At least 8 pups (N ≥ 8) under each condition as indicated were analyzed. Values in Ctl were set as 1. ∗*p* < 0.05, ∗∗*p* < 0.01, ∗∗∗*p* < 0.001 were significantly different from Ctl of the same genotype (in *C*) and TCDD exposed WT (in *F*). #*p* < 0.05, ##*p* < 0.01 was significantly different between genotypes under the same treatment condition (in *C*). DMSO, dimethyl sulfoxide; JNK, Jun N-terminal kinase; MAP3K1, mitogen-activated protein kinase kinase kinase 1; ROC, receiver operating characteristic; S1P, sphigosin-1-phosphate; TCDD, 2,3,7,8-tetrachlorodibenzo-para-dioxin.

The significant decrease of p-JNK in TCDD-exposed *Map3k1*^*+/−*^ eyelids and the fact that both the *Map3k1*^*+/−*^*Jnk1*^*−/−*^ and the *Jnk1* and *Jnk2* double gene KO mice display the EOB phenotype (20, 56) led us to postulate that JNK deficiency is responsible for the eyelid closure defects. To test this proposition, we assessed statistically the relationships between JNK activity and eyelid closure. To this effect, we measured the eyelid epithelial-specific p-JNK in embryos with normal eyelid development, under the WT, *Map3k1*^*+/−*^ and TCDD-exposed WT conditions; we also measured p-JNK in embryos associated with the open-eye defects, under *Map3k1*^*−/−*^ and TCDD-exposed *Map3k1*^*+/−*^ conditions. The collective p-JNK data and the binary scoring outcome of each eye (*i.e.*, closure or not) were subjected to receiver operating characteristic analyses in a logistic regression model. The results showed an inverse correlation between the levels of p-JNK and the possibility of eyelid closure defects (Fig. 6*A*). Specifically, the probability of developing the defects was low when p-JNK was above an arbitrary threshold, but the probability was high (>55%) when p-JNK was below the threshold. The levels of p-JNK in TCDD treated *Map3k1* heterozygous embryos fell largely below the threshold.

The S1P is a phospholipid signaling molecule that exerts biological effects by binding to and activating specific G protein–coupled S1P receptors (S1PRs) on plasma membrane (57, 58). Because the S1PR2 and 3 display redundant roles in the regulation of embryonic eyelid closure (59), we explored the possibility that the S1P signal was upstream of the MAP3K1-JNK pathway. The WT and *Map3k1*^*−/−*^ keratinocytes were treated with 20 μM S1P for different times and JNK phosphorylation was determined by Western blotting. Compared to the robust induction of p-JNK by S1P in the WT cells, the induction was significantly less, reduced by more than 50% in the *Map3k1*^*−/−*^ cells (Fig. 6, *B* and *C*), suggesting that majority of S1P signals were mediated through MAP3K1 for JNK activation.

Genetic crossing, in which genes belong to the same molecular pathways display noncomplementation for the EOB phenotype, is a powerful tool to map signaling pathways *in vivo* (20, 21). Using this approach, we investigated the relationships between S1PR, MAP3K1, and JNK1. Consistent with the observations of others (59), we found that a portion (30%) of *S1pr2/3* double KOs developed the EOB phenotypes, but neither the *S1pr2*^*+/−*^*/S1pr3*^*+/−*^ double heterozygous nor the *S1pr2*^*+/−*^*/S1pr3*^−/−^ triple allele deletion had the defects (Fig. 6*D*). The *S1pr*-mutant offspring, however, had higher incidence of the EOB phenotypes when crossed with either *Map3k1*-or *Jnk1*-KO mice. Under the *Map3k1*^+/−^ genetic conditions, 13% of *S1pr2*^*+/−*^*/S1pr3*^*+/−*^, 41% in *S1pr2*^*+/−*^*/S1pr3*^−/−^ and 100% in *S1pr2/S1pr3*-double KOs displayed the EOB defects. The *Jnk1*^+/−^ background had also increased the incidence of the EOB defects to 6% in *S1pr2*^*+/−*^*/S1pr3*^−/−^ and 100% in *S1pr2/3* double KO pups. The genetic data indicate that the S1PR-MAP3K1-JNK pathway regulates embryonic eyelid closure.

The above observations led us to speculate that, apart from *Map3k1*, other genes contributed to the pathway activity would also influence TCDD toxicity in eyelid closure. To test this prediction, we treated pregnant dams carrying *S1pr2/3*-mutant embryos and *Jnk1* gene KO with TCDD and examined the open eye phenotypes in prenatal pups. TCDD induced the open-eye defects in 11% of *S1pr2*^*+/−*^*/S1pr3*^*+/−*^ pups and 80% of *S1pr2*^*+/−*^*/S1pr3*^−/−^ pups (Fig. 6*E*). In comparison, all unexposed pups of the same genotype had closed eyelids. Similarly, the *Jnk1*^*+/−*^ and *Jnk1*^*−/−*^ mice were born with closed eyelids in the absence of TCDD exposure (60); however, they displayed an open-eye defect after *in utero* exposure to TCDD (Fig. 6*F*). The severity of the defect correlated inversely with the number of *Jnk1* allele presented in the exposed fetuses—the WT pups had normal eyelid closure, the *Jnk1* heterozygous pups had small eye openings, but the *Jnk1*^*−/−*^ pups had apparent and large eye-open defects. Thus, SRPR2/3 and JNK1 dose dependently antagonize TCDD toxicity in eyelid closure.

### The GxE interactions lead to aberrant epithelial morphogenesis and differentiation

To understand the biological consequences of the GxE interactions, we compared gene expression signatures in MAP3K1-competent (SAM HaCaT) cells and TCDD-treated MAP3K1-deficient (shRNA HaCaT) cells. The SAM cells had elevated basal JNK activity, similar to that seen in specific epithelial regions of the WT embryonic eyelids; conversely, the TCDD-treated shRNA cells likely mirrored the conditions of TCDD plus MAP3K1 deficiency. We hypothesized that the differential gene expression between shRNA-TCDD and SAM would shed light on the biological differences between WT and TCDD-treated *Map3k1*^*+/−*^ embryonic eyelids. The comparison led to the identification of approximately 24% genes differentially expressed by more than 2-fold. Among these genes, 748 (24%) were upregulated in TCDD-treated shRNA cells, whereas 2310 genes (76%) were downregulated by TCDD combined with MAP3K1 deficiency (Fig. S4*A*). When compared with the TCDD-AHR regulated genes using data derived in Fig. S2, 82 genes (11%) were found induced, and 79 (3%) genes were found repressed by TCDD combined with MAP3K1 deficiency in a manner dependent on AHR. Enrichment analyses of these genes identified keratinocyte differentiation as one of the top functions upregulated, and cell morphogenesis as the top function downregulated by TCDD plus MAP3K1 deficiency (Fig. S4, *B* and *C*).

The pathway analyses showed that either the TCDD-AHR pathway or the MAP3K1 deficiency repressed cell morphogenesis, but TCDD combined with MAP3K1 deficiency further repressed this function (Fig. S5*A*). Similarly, the morphogenetic gene expression was further decreased when the genetic and environmental factors combined (Fig. S5*B*). Some of morphogenetic genes, such as *FGFR2* and *FZD3*, have been implicated in embryonic eyelid closure (13, 61), while some others, such as *RAC3* and *NEK3*, *DKK1*, *NOG*, *SMO*, *DAB2IP, PTPRZ1*, and *GPM6A*, seem to belong to the RHO, wingless-related integration site, TGFβ/BMP, SHH, and MAPK morphogenetic pathways (10, 13, 62, 63, 64). Many other repressed genes, such as *CDH8, EFNA5/B3, EPHA4, ANOS1, TUBA1A, FLRT2 AMIGO1, POF1B, STMN1, SEMA6B*, and *WDR19*, have a role in cell adhesion, cytoskeleton reorganization, and migration.

**Figure 7:**
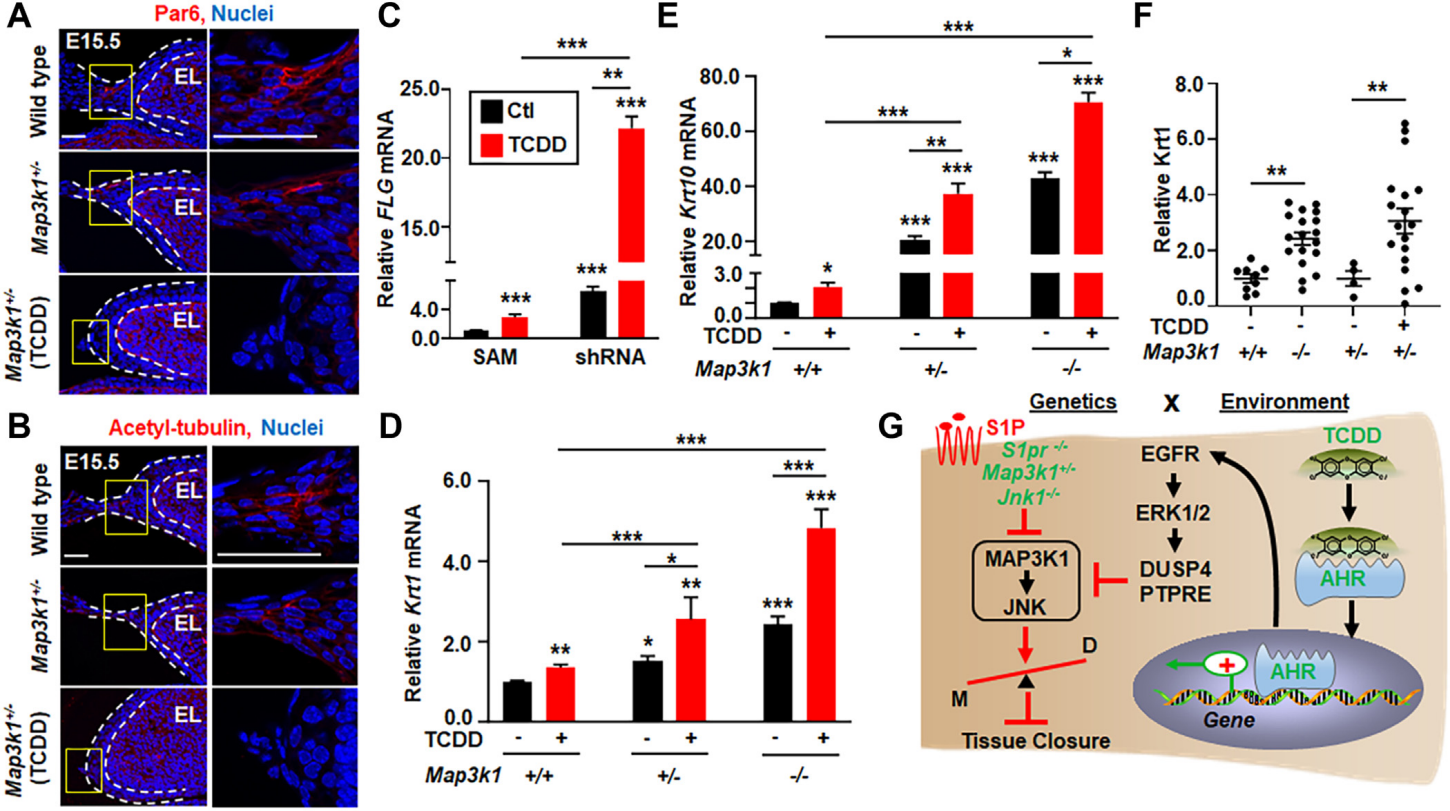
**TCDD and *Map3k1* loss-of-function disrupt cell polarity and potentiate epithelial terminal differentiation.** Eyelids of E15.5 WT, *Map3k1*^*+/−*^, and TCDD treated *Map3k1*^*+/−*^ embryos were subjected to immunohistochemistry using (*A*) anti-Par6 and (*B*) anti-acetylated-tubulin, detecting apical epithelial polarity and Hoechst labeling nuclei. *Dash lines* mark the eyelid epithelium. The scale bar represents 50 μm. *C*, the expression of *Filaggrin* (*FLG*), a marker of terminally differentiated keratinocytesin the granular and cornified epidermis, examined by qRT-PCR in shRNA HaCaT and SAM HaCaT treated with 10 nM TCDD or vehicle (DMSO, Ctl). The relative *FLG* mRNA was calculated using the housekeeping gene *GAPDH* as an internal control. *FLG* levels in SAM (Ctl) were set as 1, expression of (*D*) *Krt1* and (*E*) *Krt10*, markers of the differentiating suprabasal keratinocytes, were examined in WT, *Map3k1*^*+/−*^, and *Map3k1*^*−/−*^ keratinocytes treated with 10 nM TCDD or vehicle (DMSO). Relative expression was calculated using the housekeeping gene *GAPDH* as an internal control and compared to levels in WT keratinocytes set as 1. *F*, the E15.5 embryos of WT, *Map3k1*^*−/−*^, and *Map3k1*^*+/−*^ with or without TCDD exposure were examined by immunohistochemistry for Krt1. Staining signals were quantified, and relative expression was calculated. Data are mean ± SEM of at least three independent experiments (N ≥ 3) or at least three eye sections (N ≥ 3) per embryo and three embryos (N ≥ 3) from different litters. ∗*p* < 0.05, ∗∗*p* < 0.01, ∗∗∗*p* < 0.001 were considered statistically significant compared to WT and SAM untreated or as indicated. *G*, graphic illustration of the proposed GxE interactions model in eyelid closure defects. The environmental factor TCDD activates the AHR to induce gene expression and activate the EGFR-ERK pathways. EGFR signaling in turn induces phosphatases to inhibit the S1PR-MAP3K1-JNK pathway. This effect of TCDD is trivial and insufficient to induce the eyelid phenotype. However, in the presence of gene mutations, *i.e.*, *S1pr2/3*^*−/−*^*, Map3k1*^*+/−*^ and *Jnk1*^*−/−*^, which also slightly attenuate MAP3K1-JNK signaling, the effect of TCDD is largely amplified. As the results, the GxE interactions significantly inhibit JNK, accelerate differentiation (D) and impede morphogenesis (M) of the epithelium, leading to defective tissue closure. AHR, aryl hydrocarbon receptor; DMSO, dimethyl sulfoxide; EGFR, epidermal growth factor receptor; ERK, extracellular signal regulated kinase; JNK, Jun N-terminal kinase; Krt1, keratin 1; MAP3K1, mitogen-activated protein kinase kinase kinase 1; qRT-PCR, quantitative reverse transcription PCR; S1P, sphigosin-1-phosphate; SAM, synergistic activation mediator; TCDD, 2,3,7,8-tetrachlorodibenzo-para-dioxin.

A hallmark of epithelial morphogenesis is the establishment of cell polarity, where the Par3-Par6-αPKC complex and the acetyl-tubulin accumulate at distinct location to facilitate cell shape change, elongation, and migration (65, 66). In the embryonic eyelids, we found that the polarity markers, *i.e.*, PAR6 and acetyl-tubulin, were readily detectable in the suprabasal epithelia leading to the eyelid tip protrusion in WT and *Map3k1*^*+/−*^ E15.5 embryos. However, these markers were absent in TCDD-exposed *Map3k1*^*+/−*^ embryos (Fig. 7, *A* and *B*).

The gene expression analyses also revealed that TCDD plus MAP3K1 deficiency (shRNA-TCDD *versus* SAM) accelerated keratinocyte differentiation (Fig. S5, *C* and *D*). The differentiation genes were upregulated by TCDD-AHR or MAP3K1 deficiency, and their expression was further potentiated when the two conditions combined (Fig. S5*D*). Some of the genes, such as *S100A7* (67), *SPRR1A* (68), and *FLG* (69), are well-known markers of terminal differentiation of epithelium.

To validate the GxE effects on differentiation, we examined the expression of *FLG*, encoding a component of the epidermal differentiation complex, in the genetically modified HaCaT cells. The basal *FLG* expression in MAP3K1-deficient (shRNA) cells was 6.9-fold of that in MAP3K1-competent (SAM) cells, whereas the expression was further increased to 23.4-fold above the basal levels in shRNA cells treated with TCDD (Fig. 7*C*). The epithelial differentiation was also examined in WT, *Map3k1*^*+/−*^, and *Map3k1*^*−/−*^ cultured mouse keratinocytes. These cells maintain basal keratinocyte characteristics but undergo spontaneous terminal differentiation to the spinous epithelial cells (70). The expression of *keratin 1* (*Krt1*) and *Krt10*, products of which dimerize to form keratin intermediate filaments in the differentiated spinous epidermis, was relatively low in WT cells, but significantly increased in *Map3k1*^*+/−*^, and further elevated in *Map3k1*^*−/−*^ cells (Fig. 7, *D* and *E*). TCDD treatment also potentiated the expression of these differentiation markers. Notably, the levels of *Krt1* and *Krt10* expression in TCDD-treated *Map3k1*^*+/−*^ cells reached to the levels like that in *Map3k1*^*−/−*^ cells. Similar findings were made in the embryonic eyelids. The expression of KRT1 protein was very low in the eyelid epithelium of WT and *Map3k1*^*+/−*^ E15.5 embryos; however, the expression was increased by 2.4-fold in that of *Map3k1*^*−/−*^ embryos and 3.1-fold in TCDD-treated *Map3k1*^*+/−*^ embryos (Fig. 7*F*). Taken together, our results show that TCDD and MAP3K1 deficiency, individually and in combination, accelerate epithelial terminal differentiation and impede cell polarity in the embryonic eyelids.

## Discussion

Here, we show that TCDD activates the ocular surface epithelial AHR to impair embryonic eyelid closure in the *Map3k1*^*+/−*^ mice. Tracking the molecular footprint of TCDD, we identify TCDD-AHR-regulated gene expression, leading to a moderate yet persistent activation of the EGFR-ERK pathway and the induction of protein phosphatases, which may in turn repress JNK signaling. The JNK repression by TCDD is mild and insufficient to block eyelid closure; however, it becomes substantial in the presence of genetic perturbations. Specifically, *Map3k1* heterozygosity combined with TCDD further reduces p-JNK and causes the open-eye defects (Fig. 7*G*). We have also shown that S1P/S1PR is upstream of the MAP3K1-JNK pathway and that attenuation of the pathway activity by gene mutations, such as *S1pr2/3* and *Jnk1* KO, potentiates TCDD toxicity in eyelid morphogenesis. These results indicate that the crosstalk of genetic and environmental risk factors is mediated through an interactive signaling network, a mechanism that can likely be extrapolated to many diseases resulting from the GxE interactions.

The GxE effects are attributed largely to the crosstalk between the MAP3K1/JNK and EGFR/ERK pathways. Under certain experimental settings, ERK and JNK are known to mutually inhibit each other to maintain a balanced signaling output, which is crucial for cell fate decisions (71, 72, 73). We find a similar inhibitory relationship that extends to MAP3K1 and EGFR, upstream of JNK and ERK, respectively. As such, activation of EGFR/ERK by TCDD leads to the repression of JNK activity, whereas MAP3K1 knockdown/KO also activates EGFR/ERK and represses JNK. As the result of the feedback inhibition, trivial effects on JNK repression and ERK activation by either the TCDD or the *Map3k1* heterozygosity become significantly amplified when the two conditions are combined. Our data, together with the findings that the *Jnk1/Jnk2* double KO mice and the Ras-transgenic overexpression mice also exhibit the EOB defects (64, 74), suggest that defective eyelid closure may result from adverse imbalance of ERK/JNK signaling. Alleviating the imbalance through genetic perturbations, such as *Ahr* ^*ΔOSE*^ and *Egfr*^*ΔOSE*^, that attenuate ERK signaling, reduces TCDD-induced eyelid defects. Conversely, aggravating the imbalance through genetic ablation of *S1pr2/3* and *Jnk1* that attenuates JNK signaling potentiates the harmful effects of TCDD.

JNK signaling is an evolutionarily conserved mechanism for developmental tissue closure. While it is required for dorsal closure and thorax closure through coordinating cell shape change in *Drosophila* (75, 76), it regulates gastrulation through controlling tissue elongation in *Xenopus* (77, 78). In mice, compound knocking out the functionally redundant *Jnk1* and *Jnk2* results in defective closure of the neural tube, the optic fissure, and the eyelid (64). In this context, JNK may phosphorylate paxillin to regulate intercellular junctions interconnected with polarity (79, 80). In this work, we have additionally shown that JNK dose dependently contributes to tissue closure, as there is an inverse correlation between the number of *Jnk1* allele or the amount of p-JNK with the severity of eyelid closure defects. It is possible that other genetic and environmental insults that target JNK signaling constitute risk factors for closure abnormalities of embryonic and adult tissues.

EGFR signal is required for embryonic eyelid closure, as both the germline and OSE-specific *Egfr* KO cause basal epithelial cell hypoproliferation and a fully penetrable EOB defect (49, 81, 82). By activating the EGFR pathway, TCDD is shown to reduce the EOB phenotype of *Egfr*^*−/−*^ pups by 50%, presumably through inducing ligands that activate other receptors of EGFR family to promote lid closure (83, 84), and to promote precocious eyelid opening in postpartum pups through accelerating epithelial differentiation (85, 86, 87). Our data suggest that TCDD-induced EGFR activation is moderate and by itself is insufficient to alter the eyelid closure programs, because all TCDD-treated WT pups have normal lid closure (36). Like TCDD, *Map3k1* deficiency also induces a mild EGFR/ERK activation and epidermal differentiation. In the embryonic eyelid epithelium and cultured keratinocytes, TCDD combined with *Map3k1* deficiency have an additive or perhaps a synergistic effect on potentiating differentiation. Similar observations have been made during *in vitro* stem cell-to-epithelium differentiation, where TCDD plus *Map3k1* deficiency further accelerate basal to spinous differentiation (88). The premature differentiation may reduce flexibility of the eyelid tip epithelial cells, thereby preventing them to undergo shape change for lid closure (89). Thus, the small effects on cell activities by each agent can be exacerbated by the GxE interactions, resulting in detrimental biological outcomes.

Mouse genetics have identified over 130 genes implicated in eyelid closure (5), but these genes may regulate eyelid morphogenesis through distinct mechanisms. For example, among the EOB mutants, the *Egfr*-null mice are defective in proliferation of the basal epithelial cells (90), the c-Jun K14-KO mice have reduced EGFR activity in the eyelid tip epithelial cells (91), and the TCDD-treated *Map3k1*^*+/−*^ and the *Map3k1*^*−/−*^ eyelids have reduced JNK activity in the inferior eyelid tip epithelial cells. In these cases, the different gene products regulate temporal spatially distinct signaling and cellular activities, all of which are required for eyelid closure. On the other hand, our data have highlighted the importance of gene-gene interactions and gene-environment interactions leading to the EOB defects and suggested that genetics and the environment act as rheostats for each other to induce the defect, while each condition alone is less, or not at all, destructive. Given these findings, we predict that the monogenic, digenic, and polygenic variants that affect the AHR/EGFR/ERK-S1PR/MAP3K1/JNK network would likely increase or decrease the toxic effects of TCDD and other environmental AHR ligands on eyelid closure.

Defective embryonic eyelid closure leads to developmental abnormalities of the ocular adnexa with phenotypes comparable to human congenital disorders, such as eyelid ptosis and strabismus (9). The causes for these diseases are not fully understood but likely to be multifactorial (92, 93, 94). In this context, the risk factors and mechanisms for the eyelid closure defects revealed in this work may shed lights into the causations of relevant congenital eye diseases, as well as abnormalities of other anatomical tissue closures, such as neural tube closure, palate fusion, and ventral body wall closure, that share similar morphogenetic processes as eyelid closure. Understanding the genetic susceptibility of chemical toxicity is a crucial first step to uncover the at-risk individuals and improve personalized protection of environmental diseases.

## Experimental procedures

### Mouse strains, genotyping, and chemical treatment

The WT C57BL/6J strain and the global green fluorescence Cre reporter (*ROSA*^*mTmG*^) strain were purchased from the Jackson Laboratory; the *Map3k1*, *Jnk1*, and *S1pr2/3* mutant strains were described before (16, 55, 59). The *Egfr*^*F/F*^*, Ahr*^*F/F*^ and Le-Cre mice were gifts from Drs Threadgill, Bradfield, and Ashery-Padan, respectively (40, 41, 95). All mice were backcrossed for >12 generations to obtain congenic C57BL/6J background. Genomic DNA PCR was used to determine genotype using gene-specific primers synthesized by Integrated DNA Technologies.

*In utero* exposure was done by oral gavage treatment of the pregnant dams on gestation day 11.5 with either corn oil (vehicle) or 50 to 500 μg/kg TCDD dissolved in corn oil, as described before (36). Embryos/fetuses were harvested at E15.5 or E17.5 for histology, immunohistochemistry, and phenotype evaluation. Mice were housed in the Laboratory Animal Medical Service facility at the University of Cincinnati, College of Medicine. Experimental procedures were approved by the Institutional Animal Care and Use Committee (Protocol 23-11-01-01). All procedures with mice handling were in adherence to Guide for the Care and Use of Laboratory Animals and National Institutes of Health or Medical Research Council guidelines for animal welfare.

### Cell culture, reagents, and chemical treatment

Mouse keratinocyte lines derived from WT and *Map3k1*^*−/−*^ mice were cultured and maintained in keratinocyte serum-free medium without calcium chloride as described before (70). HaCaT, a spontaneously immortalized human keratinocyte line, was from American Type Culture Collection and cultured in Dulbecco’s modified Eagle’s medium (Gibco) supplemented with 10% fetal bovine serum, 2 mM glutamine, 1% nonessential amino acids, penicillin (100 U/ml), and streptomycin (100 μg/ml) in 5% CO_2_ at 37 °C incubator.

The MAP3K1 deficient (shRNA) HaCaT cells were generated by transduction of HaCaT cells with MAP3K1 shRNA lentiviruses. The lentiviral vectors purchased from Sigma-Aldrich (Clone ID, TRCN000000616) contain four shRNA targeting different regions of *MAP3K1* gene. To make lentivirus, the vectors were cotransfected with packaging psPAX2 (Addgene #12260) and envelope pMD2.G (Addgene #12259) plasmids into 293T packaging cells (American Type Culture Collection). HaCaT cells infected with the 4 shRNA lentiviruses were selected with puromycin (3 mg/ml) to obtain stable HaCaT shRNA cells. MAP3K1-competent HaCaT cells were generated using CRISPR/Cas9 synergistic activation mediator (SAM) system (96). Plasmids for the SAM system (*i.e.*, lenti-sgRNA (MS2) _puro, dCas9VP64, and MS2-P65-HSF1) were purchased from Addgene (Addgene, cat. #73797, 61425, and 61426). The sgRNAs for *MAP3K1* were designed based on publicly available filtering tools (https://zlab.bio/guide-design-resources) (Table S1) and cloned into lenti-sgRNA (MS2) puro. HaCaT cells were transduced with lentiviruses (*i.e.*, sgRNA, MS2, and dCas9) and selected with puromycin (3 mg/ml), blasticidin (10 mg/ml), and hygromycin (10 mg/ml) to generate stable HaCaT SAM cells. The efficiency of MAP3K1 knockdown and overexpression was validated by examination of gene expression at mRNA and protein level.

Cells were treated with 10 nM TCDD in the presence or absence of 10 μM CH-223191 for 24 h or with 10 μM AG1478, 10 to 20 μM PD98059, and SP600125 for 8 h or with 20 μM S1P for different times and harvested for RNA and protein analyses. Cell culture media, reagents, chemicals, and inhibitors are listed in Table S2.

### Phenotype evaluation and immunohistochemistry

E17.5 fetuses were harvested, and the eyes were photographed with a Leica MZ-16FA dissecting microscope. The size of the eye open was determined by tracing the edge of the eyelids and measuring areas with ImageJ software (https://imagej.net/ij/). Pixel values were transferred to actual areas (mm^2^) based on the scale information of each image. The value is zero if the eyelids are fully closed.

For immunohistochemistry, E15.5 embryos were harvested and fixed with 4% paraformaldehyde at 4 °C overnight, followed by embedding in optimal cutting temperature compound and freezing. Eye sections were subjected to immunohistochemistry with specific primary antibodies and fluorescent labeled secondary antibodies. Primary and secondary antibodies are listed in Table S3. Images were captured by the Zeiss Axioplan 2 fluorescence and confocal microscope equipped with AxioVision software (https://www.micro-shop.zeiss.com/en/us/system/software-axiovision+software-products/1007/). For semiquantification of the fluorescent signals, the E-cadherin-labeled epithelial cells were used to mark the suprafacial layer; the region of interest (ROI) in mucocutaneous junction area were outlined and levels of p-JNK or p-ERK were determined with the ImageJ software. The blank region next to the ROI was considered as background. The relative p-JNK and p-ERK were calculated after mean intensity (total intensity/area) of the ROI subtracting the background. The levels were compared to those in untreated WT samples, set as 1.

For the threshold model, measurement of p-JNK and the eye-open or close statuses were subjected to a logistic regression model based on the binary outcome of eyelid closure with receiver operating characteristic analyses.

### Western blotting

Cells were lysed in RIPA buffer containing 20 mM Tris–HCl, pH 7.5, 150 mm NaCl, 1 mM EDTA, 1% Nonidet P-40, 0.5% sodium deoxycholate, 10 μg/ml aprotinin, 10 μg/ml leupeptin, 1 mM Na_3_VO_4_, and 100 μM phenylmethylsulphonyl fluoride. Lysates (50–100 μg protein) were subjected to SDS-PAGE. Proteins were transferred to nitrocellulose membranes and incubated with primary and horseradish peroxidase-secondary antibodies. Antibodies for Western blotting are listed in Table S3. The chemiluminescence signals were captured with UVP Biochemi System (G58-bc-042704 Darkroom Imager). Data were quantified with ImageJ using densitometry normalized with β-actin as loading control. Specifically, background exposure values were subtracted from the values of the protein band of interest and the loading control, *e.g.* β-actin, the ratio of adjusted values of protein *versus* loading control was calculated, and normalized to that in Ctl, TCDD treated or SAM cells, set as 1.

### RNA isolation, reverse transcription, and RT-PCR

Total RNA was isolated using PureLink RNA Mini Kit (12183025, Invitrogen). First-strand complementary DNAs were synthesized by 0.5 μg of total RNA using SuperScript IV Reverse Transcriptase (Invitrogen) according to the manufacturer’s instruction. PCR reactions were carried out with Stratagene Mx3000P RT-PCR system (Agilent Technologies). Power SYBR Green PCR Master Mix (Applied Biosystems) was used as the detection format. The reaction was heated to 95 °C for 10 min, followed by 40 cycles of denaturation at 95 °C for 15 s and annealing elongation at 60 °C for 60 s. Gene expression was calculated by comparative ΔΔCt-method normalized to a constitutively expressed housekeeping gene (*GADPH*). Data represent results of triplicates in at least three independent experiments. The sequences of PCR primers are listed in Table S4.

### RNA-seq

RNA quality was QC analyzed by Bioanalyzer (Agilent Technologies). RNA sequencing of biological triplicate samples was performed by the Genomics, Epigenomics, and Sequencing Core at the University of Cincinnati using established protocols described previously (70). Details were in the Supplementary Materials and Methods. Raw data and processed files were either uploaded to NCBI GEO database (GSE237130) and in the process of submission.

### Transcriptome analyses

To identify genes regulated by the TCDD-AHR pathways, we compare differential gene expressions in HaCaT cells treated for overnight with (1) vehicle, (2) 10 nM TCDD, (3) 10 μM AHR inhibitor CH223191, and (4) TCDD plus CH223191. Gene expression in samples (2) were compared with combined samples in (1), (3) and (4) to identify significantly differential expressed genes dependent on TCDD-AHR (3600 genes) (padj < 0.05). The TCDD-AHR dependent genes were submitted to QIAGEN ingenuity pathway analyses for pathway analysis, Upstream Regulators and Causal Networks. Heat maps were generated by using Heatmapper (97).

To identify genes affected by MAP3K1 loss-of-functions, we compared gene expressions in shRNA *versus* SAM cells. The upregulated (log2 fold change >1, padj < 0.05) and downregulated (log2 fold change <-1, padj < 0.05) genes were subjected to functional enrichment analyses with the Metascape software (v3.5.20230501, https://metascape.org/gp/index.html#/main/step1) (98). To analyze the gene expression signatures of the gene-environment interactions, we compared expression in TCDD-treated shRNA HaCaT with untreated SAM HaCaT; the data were further cross compared with the previously identified 3600 TCDD-AHR dependent genes, and the overlapping differential expressed genes were selected for functional enrichment analysis using Metascape.

### Data quantification and statistical analysis

Quantification of the eye open phenotype, immunohistochemistry positive signals and immunoblotting band intensity were done by Image J software (ver. Fiji-2.1.1). All data are shown as mean ± standard error of the mean (SEM) based on at least three independent experiments and analyzed using Student’s two-tailed *t* test or One-way analysis of variance following Dunnett’s test for multiple comparisons. #*p*, ∗*p* < 0.05; ##*p*, ∗∗*p* < 0.01; and ∗∗∗*p* < 0.001 are considered statistically significant.

## Data availability

All relevant data can be found within the article and its supporting information.

## Supporting information

This article contains supporting information.

## Conflict of interest

The authors declare that they have no conflicts of interest with the contents of this article.

## References

[1] Kuida K., Boucher D.M. (2004). Functions of MAP kinases: insights from gene-targeting studies. J. Biochem..

[2] Hagemann C., Blank J.L. (2001). The ups and downs of MEK kinase interactions. Cell Signal..

[3] Uhlik M.T., Abell A.N., Cuevas B.D., Nakamura K., Johnson G.L. (2004). Wiring diagrams of MAPK regulation by MEKK1, 2, and 3. Biochem. Cell Biol..

[4] Craig E.A., Stevens M.V., Vaillancourt R.R., Camenisch T.D. (2008). MAP3Ks as central regulators of cell fate during development. Dev. Dyn..

[5] Wang J., Kimura E., Mongan M., Xia Y. (2021). Genetic control of MAP3K1 in eye development and sex differentiation. Cells.

[6] Harris M.J., Juriloff D.M. (1986). Eyelid development and fusion induced by cortisone treatment in mutant, lidgap-Miller, foetal mice. A scanning electron microscope study. J. Embryol. Exp. Morphol..

[7] Findlater G.S., McDougall R.D., Kaufman M.H. (1993). Eyelid development, fusion and subsequent reopening in the mouse. J. Anat..

[8] Teramoto S., Fujii S., Yoshida A., Shirasu Y. (1988). Morphological and genetic characteristics of the open-eyelid mutant spontaneously occurring in NC-strain mice. Jikken Dobutsu.

[9] Meng Q., Mongan M., Carreira V., Kurita H., Liu C.Y., Kao W.W. (2014). Eyelid closure in embryogenesis is required for ocular adnexa development. Invest. Ophthalmol. Vis. Sci..

[10] Gage P.J., Qian M., Wu D., Rosenberg K.I. (2008). The canonical Wnt signaling antagonist DKK2 is an essential effector of PITX2 function during normal eye development. Dev. Biol..

[11] Schaeper U., Vogel R., Chmielowiec J., Huelsken J., Rosario M., Birchmeier W. (2007). Distinct requirements for Gab1 in Met and EGF receptor signaling in vivo. Proc. Natl. Acad. Sci. U. S. A..

[12] Qu C.K., Yu W.M., Azzarelli B., Feng G.S. (1999). Genetic evidence that Shp-2 tyrosine phosphatase is a signal enhancer of the epidermal growth factor receptor in mammals. Proc. Natl. Acad. Sci. U. S. A..

[13] Huang J., Dattilo L.K., Rajagopal R., Liu Y., Kaartinen V., Mishina Y. (2009). FGF-regulated BMP signaling is required for eyelid closure and to specify conjunctival epithelial cell fate. Development.

[14] Tao H., Ono K., Kurose H., Noji S., Ohuchi H. (2006). Exogenous FGF10 can rescue an eye-open at birth phenotype of Fgf10-null mice by activating activin and TGFalpha-EGFR signaling. Dev. Growth Differ..

[15] Yujiri T., Fanger G.R., Garrington T.P., Schlesinger T.K., Gibson S., Johnson G.L. (1999). MEK kinase 1 (MEKK1) transduces c-Jun NH2-terminal kinase activation in response to changes in the microtubule cytoskeleton. J. Biol. Chem..

[16] Zhang L., Wang W., Hayashi Y., Jester J.V., Birk D.E., Gao M. (2003). A role for MEK kinase 1 in TGF-beta/activin-induced epithelium movement and embryonic eyelid closure. EMBO J..

[17] Juriloff D.M., Harris M.J., Mah D.G. (2005). The open-eyelid mutation, lidgap-Gates, is an eight-exon deletion in the mouse Map3k1 gene. Genomics.

[18] Parker A., Cross S.H., Jackson I.J., Hardisty-Hughes R., Morse S., Nicholson G. (2015). The goya mouse mutant reveals distinct newly identified roles for MAP3K1 in the development and survival of cochlear sensory hair cells. Dis. Model. Mech..

[19] Zhang L., Deng M., Parthasarathy R., Wang L., Mongan M., Molkentin J.D. (2005). MEKK1 transduces activin signals in keratinocytes to induce actin stress fiber formation and migration. Mol. Cell Biol..

[20] Takatori A., Geh E., Chen L., Zhang L., Meller J., Xia Y. (2008). Differential transmission of MEKK1 morphogenetic signals by JNK1 and JNK2. Development.

[21] Yook K.J., Proulx S.R., Jorgensen E.M. (2001). Rules of nonallelic noncomplementation at the synapse in Caenorhabditis elegans. Genetics.

[22] Chapin R.E., Robbins W.A., Schieve L.A., Sweeney A.M., Tabacova S.A., Tomashek K.M. (2004). Off to a good start: the influence of pre- and periconceptional exposures, parental fertility, and nutrition on children's health. Environ. Health Perspect..

[23] Peters J.M., Narotsky M.G., Elizondo G., Fernandez-Salguero P.M., Gonzalez F.J., Abbott B.D. (1999). Amelioration of TCDD-induced teratogenesis in aryl hydrocarbon receptor (AhR)-null mice. Toxicol. Sci..

[24] Fernandez-Salguero P.M., Hilbert D.M., Rudikoff S., Ward J.M., Gonzalez F.J. (1996). Aryl-hydrocarbon receptor-deficient mice are resistant to 2,3,7,8-tetrachlorodibenzo-p-dioxin-induced toxicity. Toxicol. Appl. Pharmacol..

[25] Hankinson O. (1995). The aryl hydrocarbon receptor complex. Annu. Rev. Pharmacol. Toxicol..

[26] Fujii-Kuriyama Y., Mimura J. (2005). Molecular mechanisms of AhR functions in the regulation of cytochrome P450 genes. Biochem. Biophys. Res. Commun..

[27] Puga A., Barnes S.J., Dalton T.P., Chang C., Knudsen E.S., Maier M.A. (2000). Aromatic hydrocarbon receptor interaction with the retinoblastoma protein potentiates repression of E2F-dependent transcription and cell cycle arrest. J. Biol. Chem..

[28] Elferink C.J. (2003). Aryl hydrocarbon receptor-mediated cell cycle control. Prog. Cell Cycle Res..

[29] Hurst C.H., Abbott B.D., DeVito M.J., Birnbaum L.S. (1998). 2,3,7,8-Tetrachlorodibenzo-p-dioxin in pregnant Long Evans rats: disposition to maternal and embryo/fetal tissues. Toxicol. Sci..

[30] Abbott B.D. (1995). Review of the interaction between TCDD and glucocorticoids in embryonic palate. Toxicology.

[31] Abbott B.D., Probst M.R., Perdew G.H., Buckalew A.R. (1998). AH receptor, ARNT, glucocorticoid receptor, EGF receptor, EGF, TGF alpha, TGF beta 1, TGF beta 2, and TGF beta 3 expression in human embryonic palate, and effects of 2,3,7,8-tetrachlorodibenzo-p-dioxin (TCDD). Teratology.

[32] Couture L.A., Abbott B.D., Birnbaum L.S. (1990). A critical review of the developmental toxicity and teratogenicity of 2,3,7,8-tetrachlorodibenzo-p-dioxin: recent advances toward understanding the mechanism. Teratology.

[33] Yoshioka W., Peterson R.E., Tohyama C. (2011). Molecular targets that link dioxin exposure to toxicity phenotypes. J. Steroid Biochem. Mol. Biol..

[34] Thomae T.L., Stevens E.A., Liss A.L., Drinkwater N.R., Bradfield C.A. (2006). The teratogenic sensitivity to 2,3,7,8-tetrachlorodibenzo-p-dioxin is modified by a locus on mouse chromosome 3. Mol. Pharmacol..

[35] Keller J.M., Zelditch M.L., Huet Y.M., Leamy L.J. (2008). Genetic differences in sensitivity to alterations of mandible structure caused by the teratogen 2,3,7,8-tetrachlorodibenzo-p-dioxin. Toxicol. Pathol..

[36] Mongan M., Meng Q., Wang J., Kao W.W., Puga A., Xia Y. (2015). Gene-environment interactions target mitogen-activated protein 3 kinase 1 (MAP3K1) signaling in eyelid morphogenesis. J. Biol. Chem..

[37] Anderson A.L., Dubanksy B.D., Wilson L.B., Tanguay R.L., Rice C.D. (2022). Development and applications of a zebrafish (Danio rerio) CYP1A-targeted monoclonal antibody (CRC4) with reactivity across vertebrate taxa: evidence for a conserved CYP1A epitope. Toxics.

[38] Nebert D.W., Roe A.L., Dieter M.Z., Solis W.A., Yang Y., Dalton T.P. (2000). Role of the aromatic hydrocarbon receptor and [Ah] gene battery in the oxidative stress response, cell cycle control, and apoptosis. Biochem. Pharmacol..

[39] Omiecinski C.J., Redlich C.A., Costa P. (1990). Induction and developmental expression of cytochrome P450IA1 messenger RNA in rat and human tissues: detection by the polymerase chain reaction. Cancer Res..

[40] shery-Padan R., Marquardt T., Zhou X., Gruss P. (2000). Pax6 activity in the lens primordium is required for lens formation and for correct placement of a single retina in the eye. Genes Dev..

[41] Walisser J.A., Bunger M.K., Glover E., Bradfield C.A. (2004). Gestational exposure of Ahr and Arnt hypomorphs to dioxin rescues vascular development. Proc. Natl. Acad. Sci. U. S. A..

[42] Ema M., Ohe N., Suzuki M., Mimura J., Sogawa K., Ikawa S. (1994). Dioxin binding activities of polymorphic forms of mouse and human arylhydrocarbon receptors. J. Biol. Chem..

[43] Hoffer A., Chang C.Y., Puga A. (1996). Dioxin induces transcription of fos and jun genes by Ah receptor-dependent and -independent pathways. Toxicol. Appl. Pharmacol..

[44] Boukamp P., Petrussevska R.T., Breitkreutz D., Hornung J., Markham A., Fusenig N.E. (1988). Normal keratinization in a spontaneously immortalized aneuploid human keratinocyte cell line. J. Cell Biol..

[45] Sartor M.A., Schnekenburger M., Marlowe J.L., Reichard J.F., Wang Y., Fan Y. (2009). Genomewide analysis of aryl hydrocarbon receptor binding targets reveals an extensive array of gene clusters that control morphogenetic and developmental programs. Environ. Health Perspect..

[46] Kramer A., Green J., Pollard J., Tugendreich S. (2014). Causal analysis approaches in ingenuity pathway analysis. Bioinformatics.

[47] Thompson D.M., Gill G.N. (1985). The EGF receptor: structure, regulation and potential role in malignancy. Cancer Surv..

[48] Martinelli E., Morgillo F., Troiani T., Ciardiello F. (2017). Cancer resistance to therapies against the EGFR-RAS-RAF pathway: the role of MEK. Cancer Treat Rev..

[49] Miettinen P.J., Berger J.E., Meneses J., Phung Y., Pedersen R.A., Werb Z. (1995). Epithelial immaturity and multiorgan failure in mice lacking epidermal growth factor receptor. Nature.

[50] Aguilar J.L., Kulkarni R., Randis T.M., Soman S., Kikuchi A., Yin Y. (2009). Phosphatase-dependent regulation of epithelial mitogen-activated protein kinase responses to toxin-induced membrane pores. PLoS One.

[51] Chen Y.R., Tan T.H. (1998). Inhibition of the c-Jun N-terminal kinase (JNK) signaling pathway by curcumin. Oncogene.

[52] Ip Y.T., Davis R.J. (1998). Signal transduction by the c-Jun N-terminal kinase (JNK)--from inflammation to development. Curr. Opin. Cell Biol..

[53] Akimoto M., Mishra K., Lim K.T., Tani N., Hisanaga S.I., Katagiri T. (2009). Protein tyrosine phosphatase epsilon is a negative regulator of FcepsilonRI-mediated mast cell responses. Scand. J. Immunol..

[54] Xu W., Nie C., Chen X. (2023). DUSP4 inhibits autophagic cell death and apoptosis in colorectal cancer by regulating BCL2-Beclin1/Bax signaling. Mol. Biol. Rep..

[55] Xia Y., Makris C., Su B., Li E., Yang J., Nemerow G.R. (2000). MEK kinase 1 is critically required for c-Jun N-terminal kinase activation by proinflammatory stimuli and growth factor-induced cell migration. Proc. Natl. Acad. Sci. U. S. A..

[56] Weston C.R., Wong A., Hall J.P., Goad M.E., Flavell R.A., Davis R.J. (2004). The c-Jun NH2-terminal kinase is essential for epidermal growth factor expression during epidermal morphogenesis. Proc. Natl. Acad. Sci. U. S. A..

[57] Sternweis P.C., Carter A.M., Chen Z., Danesh S.M., Hsiung Y.F., Singer W.D. (2007). Regulation of Rho guanine nucleotide exchange factors by G proteins. Adv. Protein Chem..

[58] Aarthi J.J., Darendeliler M.A., Pushparaj P.N. (2011). Dissecting the role of the S1P/S1PR axis in health and disease. J. Dent. Res..

[59] Herr D.R., Lee C.W., Wang W., Ware A., Rivera R., Chun J. (2013). Sphingosine 1-phosphate receptors are essential mediators of eyelid closure during embryonic development. J. Biol. Chem..

[60] Kuan C.Y., Yang D.D., Samanta Roy D.R., Davis R.J., Rakic P., Flavell R.A. (1999). The Jnk1 and Jnk2 protein kinases are required for regional specific apoptosis during early brain development. Neuron.

[61] Fuhrmann S. (2008). Wnt signaling in eye organogenesis. Organogenesis.

[62] Geh E., Meng Q., Mongan M., Wang J., Takatori A., Zheng Y. (2011). Mitogen-activated protein kinase kinase kinase 1 (MAP3K1) integrates developmental signals for eyelid closure. Proc. Natl. Acad. Sci. U. S. A..

[63] Kuracha M.R., Siefker E., Licht J.D., Govindarajan V. (2013). Spry1 and Spry2 are necessary for eyelid closure. Dev. Biol..

[64] Weston C.R., Wong A., Hall J.P., Goad M.E., Flavell R.A., Davis R.J. (2003). JNK initiates a cytokine cascade that causes Pax2 expression and closure of the optic fissure. Genes Dev..

[65] Gibson M.C., Perrimon N. (2003). Apicobasal polarization: epithelial form and function. Curr. Opin. Cell Biol..

[66] Chen J., Zhang M. (2013). The Par3/Par6/aPKC complex and epithelial cell polarity. Exp. Cell Res..

[67] Kizawa K., Takahara H., Unno M., Heizmann C.W. (2011). S100 and S100 fused-type protein families in epidermal maturation with special focus on S100A3 in mammalian hair cuticles. Biochimie.

[68] Heikinheimo K., Kurppa K.J., Laiho A., Peltonen S., Berdal A., Bouattour A. (2015). Early dental epithelial transcription factors distinguish ameloblastoma from keratocystic odontogenic tumor. J. Dent. Res..

[69] Hoober J.K., Eggink L.L. (2022). The Discovery and function of Filaggrin. Int. J. Mol. Sci..

[70] Wang J., Mongan M., Zhang X., Xia Y. (2021). Isolation and long-term expansion of murine epidermal stem-like cells. PLoS One.

[71] Junttila M.R., Li S.P., Westermarck J. (2008). Phosphatase-mediated crosstalk between MAPK signaling pathways in the regulation of cell survival. FASEB J..

[72] Bluthgen N., Legewie S. (2008). Systems analysis of MAPK signal transduction. Essays Biochem..

[73] Dong Z., Bode A.M. (2003). Dialogue between ERKs and JNKs: friendly or antagonistic?. Mol. Interv..

[74] Burgess D., Zhang Y., Siefker E., Vaca R., Kuracha M.R., Reneker L. (2010). Activated Ras alters lens and corneal development through induction of distinct downstream targets. BMC Dev. Biol..

[75] Jacinto A., Wood W., Balayo T., Turmaine M., Martinez-Arias A., Martin P. (2000). Dynamic actin-based epithelial adhesion and cell matching during Drosophila dorsal closure. Curr. Biol..

[76] Martin-Blanco E., Pastor-Pareja J.C., Garcia-Bellido A. (2000). JNK and decapentaplegic signaling control adhesiveness and cytoskeleton dynamics during thorax closure in Drosophila. Proc. Natl. Acad. Sci. U. S. A..

[77] Carron C., Bourdelas A., Li H.Y., Boucaut J.C., Shi D.L. (2005). Antagonistic interaction between IGF and Wnt/JNK signaling in convergent extension in Xenopus embryo. Mech. Dev..

[78] Kim G.H., Han J.K. (2005). JNK and ROKalpha function in the noncanonical Wnt/RhoA signaling pathway to regulate Xenopus convergent extension movements. Dev. Dyn..

[79] Huang C., Rajfur Z., Borchers C., Schaller M.D., Jacobson K. (2003). JNK phosphorylates paxillin and regulates cell migration. Nature.

[80] You H., Lei P., Andreadis S.T. (2013). JNK is a novel regulator of intercellular adhesion. Tissue Barriers.

[81] Hansen L.A., Alexander N., Hogan M.E., Sundberg J.P., Dlugosz A., Threadgill D.W. (1997). Genetically null mice reveal a central role for epidermal growth factor receptor in the differentiation of the hair follicle and normal hair development. Am. J. Pathol..

[82] Sibilia M., Wagner E.F. (1995). Strain-dependent epithelial defects in mice lacking the EGF receptor. Science.

[83] Miettinen H.M., Huuskonen H., Partanen A.M., Miettinen P., Tuomisto J.T., Pohjanvirta R. (2004). Effects of epidermal growth factor receptor deficiency and 2,3,7,8-tetrachlorodibenzo-p-dioxin on fetal development in mice. Toxicol. Lett..

[84] Patel R.D., Kim D.J., Peters J.M., Perdew G.H. (2006). The aryl hydrocarbon receptor directly regulates expression of the potent mitogen epiregulin. Toxicol. Sci..

[85] Sutter C.H., Bodreddigari S., Campion C., Wible R.S., Sutter T.R. (2011). 2,3,7,8-Tetrachlorodibenzo-p-dioxin increases the expression of genes in the human epidermal differentiation complex and accelerates epidermal barrier formation. Toxicol. Sci..

[86] Kennedy L.H., Sutter C.H., Leon Carrion S., Tran Q.T., Bodreddigari S., Kensicki E. (2013). 2,3,7,8-Tetrachlorodibenzo-p-dioxin-mediated production of reactive oxygen species is an essential step in the mechanism of action to accelerate human keratinocyte differentiation. Toxicol. Sci..

[87] Sutter C.H., Rainwater H.M., Sutter T.R. (2020). Contributions of nitric oxide to AHR-ligand-mediated keratinocyte differentiation. Int. J. Mol. Sci..

[88] Wang J., Xiao B., Kimura E., Mongan M., Xia Y. (2022). The combined effects of Map3k1 mutation and dioxin on differentiation of keratinocytes derived from mouse embryonic stem cells. Sci. Rep..

[89] Byrne C., Tainsky M., Fuchs E. (1994). Programming gene expression in developing epidermis. Development.

[90] Threadgill D.W., Dlugosz A.A., Hansen L.A., Tennenbaum T., Lichti U., Yee D. (1995). Targeted disruption of mouse EGF receptor: effect of genetic background on mutant phenotype. Science.

[91] Zenz R., Scheuch H., Martin P., Frank C., Eferl R., Kenner L. (2003). c-Jun regulates eyelid closure and skin tumor development through EGFR signaling. Dev. Cell.

[92] Lorenz B. (2002). Genetics of isolated and syndromic strabismus: facts and perspectives. Strabismus.

[93] Oystreck D.T., Morales J., Chaudhry I., Alorainy I.A., Elkhamary S.M., Pasha T.M. (2012). Visual loss in orbitofacial neurofibromatosis type 1. Ophthalmology.

[94] Finsterer J. (2003). Ptosis: causes, presentation, and management. Aesthet. Plast. Surg..

[95] Maklad A., Nicolai J.R., Bichsel K.J., Evenson J.E., Lee T.C., Threadgill D.W. (2009). The EGFR is required for proper innervation to the skin. J. Invest. Dermatol..

[96] Konermann S., Brigham M.D., Trevino A.E., Joung J., Abudayyeh O.O., Barcena C. (2015). Genome-scale transcriptional activation by an engineered CRISPR-Cas9 complex. Nature.

[97] Babicki S., Arndt D., Marcu A., Liang Y., Grant J.R., Maciejewski A. (2016). Heatmapper: web-enabled heat mapping for all. Nucleic Acids Res..

[98] Zhou Y., Zhou B., Pache L., Chang M., Khodabakhshi A.H., Tanaseichuk O. (2019). Metascape provides a biologist-oriented resource for the analysis of systems-level datasets. Nat. Commun..

