# Supplementary material for: Crosstalk of MAP3K1 and EGFR signaling mediates gene-environment interactions that block developmental tissue closure
Source: J Biol Chem. 2024 Jun 18;300(7):107486. doi: 10.1016/j.jbc.2024.107486 (PMC11294703; doi:10.1016/j.jbc.2024.107486)
Supplement: Supporting information [file mmc1.pdf]

Crosstalk of MAP3K1 and EGFR signaling mediates gene-environment interactions that block developmental tissue closure

Jingjing Wang[1,2], Bo Xiao[1], Eiki Kimura[3], Maureen Mongan, Wei-Wen Hsu, Mario Medvedovic, Alvaro Puga, Ying Xia[*]

Department of Environmental and Public Health Sciences, University of Cincinnati, College of Medicine, Cincinnati, Ohio, 45267, USA

[1] Authors contribute equally to the work

[2] Current address: Molecular Medicine Research Institute, 428 Oakmead Parkway, Sunnyvale, CA 94085

[3] Current address: Department of Environmental Health, University of Fukui School of Medical Sciences

*Correspondence Should Be Addressed To:

Ying Xia, PhD

Department of Environmental Health

University of Cincinnati, College of Medicine

160 Panzeca Way

Cincinnati, OH 45267-0056

Mail Location: 0056

Email, ying.xia@uc.edu

Supporting Information

Supplementary Materials and Methods

RNA sequencing. Poly (A) RNA was isolated from 1 µg total RNA using the NEBNext Poly(A) mRNA Magnetic Isolation Module (New England BioLabs, Ipswich, MA), and enriched with SMARTer Apollo automated NGS library prep system (Takara Bio USA, Mountain View, CA). The cDNA library was prepared using the NEBNext Ultra II Directional RNA Library Prep kit (New England BioLabs) PCR cycle number of 8, followed by library QC and quantification via real-time qPCR (NEBNext Library Quant Kit, New England BioLabs). The individually indexed libraries were proportionally pooled and loaded on a NextSeq 550 sequencer (Illumina, San Diego, CA) under the sequencing setting of single read 1x85 bp. Once sequencing is completed, adapter trimmed fastq files for downstream data analyses were generated via Illumina BaseSpace Sequence Hub. A total of 23.4 +/- 1.6 (mean +/- SE) million pass filter reads per sample were generated. For each sample, >97% reads were aligned to HG38 reference genome, including >99% of stranded sequences, and ~85% reads aligned to coding and untranslated region (UTR), which indicated good RNA and data quality. General bioinformatics were performed using BaseSpace app RNA-Seq Alignment app v2.0.2 with Mark Duplicates unchecked, followed by RNA-seq Differential Expression app version 1.0.1. The analyses used Salmon for quantification (counts and transcripts per million, TPM), followed by DESeq2 to identify differentially expressed genes. Significant genes were selected based on adjusted *p*-value <0.05.

Supplementary Figures and Legends

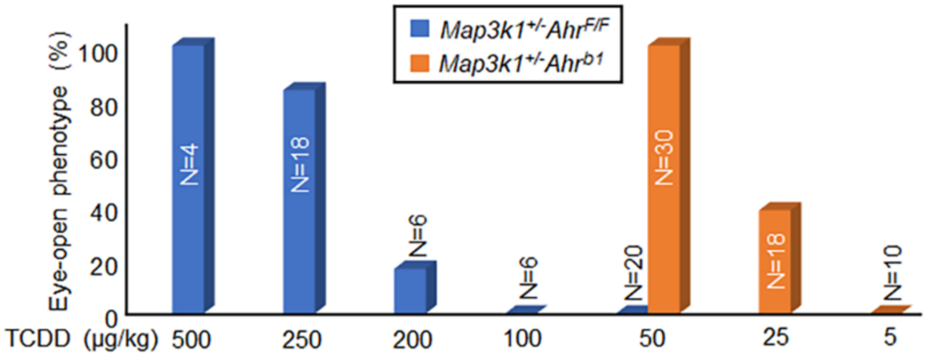

Figure S1. Strain-specific TCDD toxicity. Pregnant dams carrying *Map3k1*$^{+/-}$ embryos with *Ahr*^{b1}, encoding an AHR of high ligand-binding affinity, and *Ahr*^{F}, encoding an AHR of low ligand-binding affinity, were exposed to different doses of TCDD at E11.5. The E17.5 fetuses were examined for the eye-open phenotype. Number of pups with the open-eye defects versus number of total pups (N) of each genetic and exposure condition was calculated.

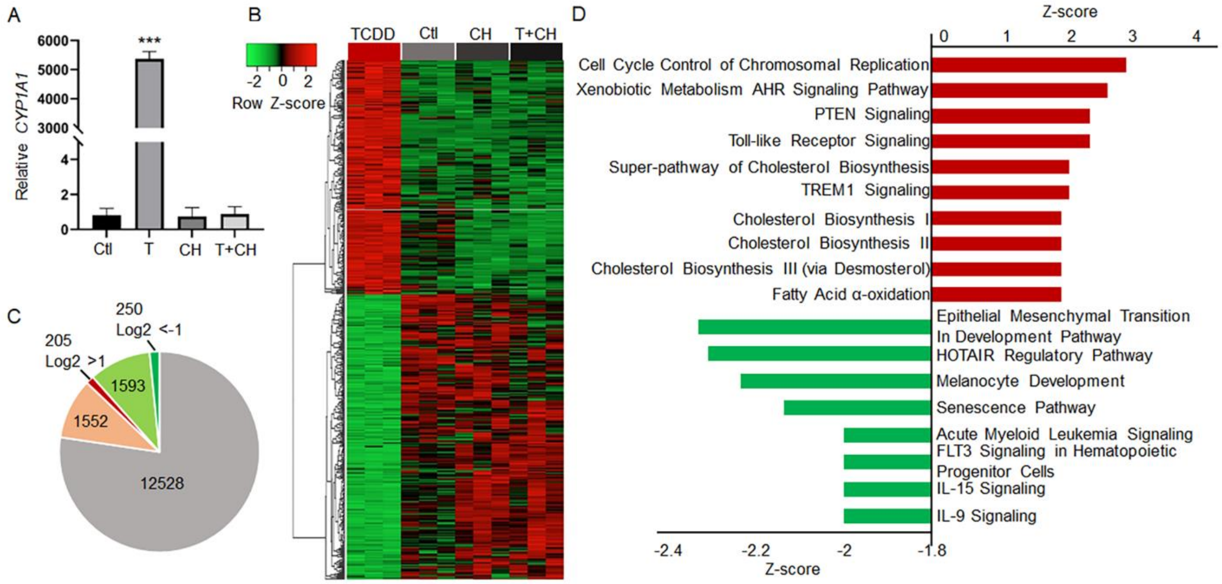

Figure S2. The TCDD-AHR axis regulated transcriptomes in HaCaT cells. (**A**) *CYP1A1* expression was examined by qRT-PCR in HaCaT cells treated with vehicle (DMSO, Ctl), TCDD (T, 10 nM), CH223191 (CH, 10 µM), and TCDD plus CH223191 (T+CH). Relative *CYP1A1* expression was calculated using *GAPDH* expression as an internal control. ***p<0.001 was considered significantly different from the Ctl samples, set as 1. (**B**) Heatmap of the TCDD-AHR dependent gene expression, marking significantly up-regulated (red) and down-regulated (green) genes. (**C**) Out of more than 12 k genes, 3600 genes were significantly up- (red) or down- (green) regulated by TCDD treatment. Among these genes, 455 gene were differentially expressed by 2-fold in a manner dependent on AHR. (**D**) Functional enrichment analysis of the 455 differentially expressed genes using Metascape.

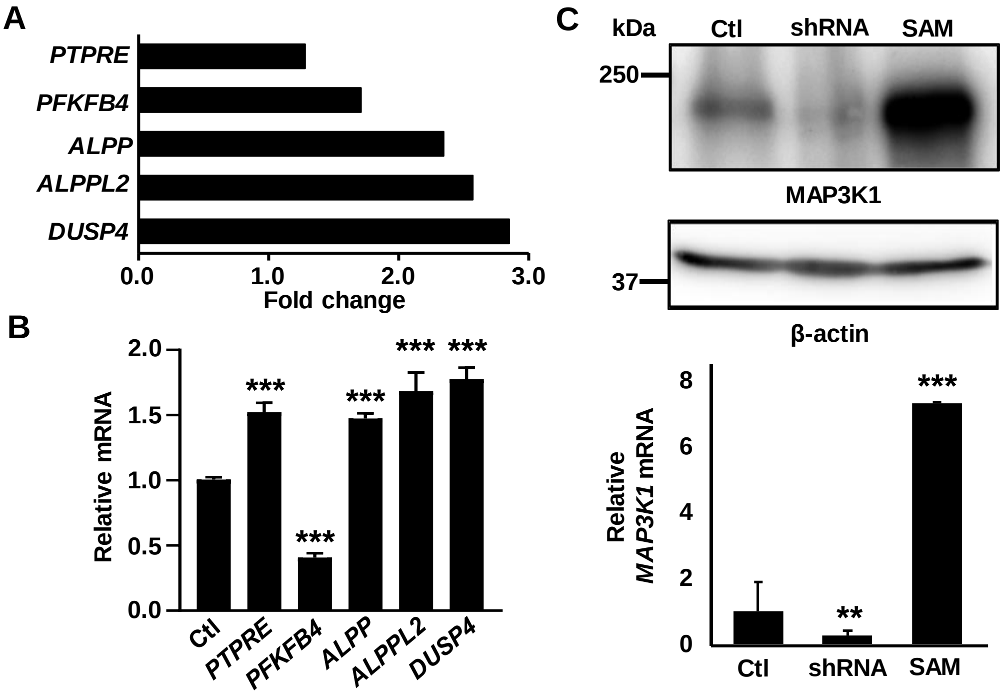

Figure S3. TCDD-AHR regulated phosphatase expression, and genetically modified HaCaT cells with differential MAP3K1 expression. (**A**) The RNA-seq data revealed TCDD-AHR dependent expression of selective phosphatases, including *PTPRE*, *PFKFB4*, *ALPP*, *ALPPL2* and *DUSP4*. (**B**) qRT-PCR examination of phosphatase gene expression in HaCaT cells treated with vehicle (DMSO, Ctl) or TCDD (10 nM). Relative mRNA was calculated in comparison to that of the housekeeping gene *GAPDH*. (**C**) HaCaT cells and their genetically modified derivatives, i.e., shRNA (MAP3K1 knockdown); and SAM (MAP3K1 overexpression) were examined by Western blotting for MAP3K1 and β-actin, and by qRT-PCR for *MAP3K1* and *GAPDH*. Compared to HaCaT cells (Ctl), shRNA cells had significantly decreased *MAP3K1* expression, whereas SAM cells had a significantly increased *MAP3K1* expression. Data were shown as mean ± SEM based on at least three independent experiments (N≥3). $^{**}p<0.01$ and $^{***}p<0.001$ are considered significantly different compared to values in Ctl set as 1.

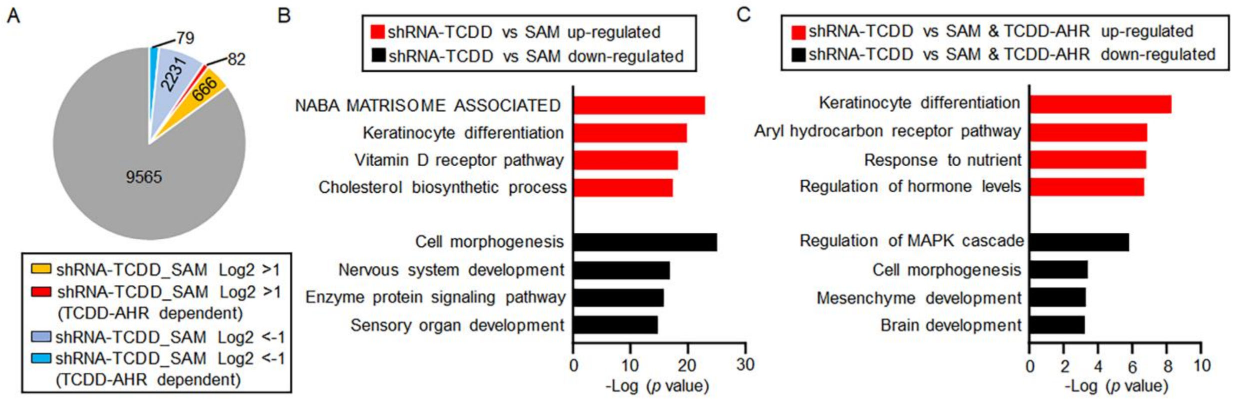

Figure S4. The effect of GxE interactions on gene expression. (**A**) Comparison of significant genes (FDR < 0.1) in the transcriptome data of TCDD-treated shRNA and control-SAM cells identified the GxE interactions up- and down-regulated genes by 2-fold, and genes dependent on TCDD-AHR. (**B**) Genes up- and down-regulated by the GxE interactions and (**C**) those also dependent on AHR were subject to gene enrichment analysis using Metascape. The top biological functions of up-regulated (red bar) and down-regulated (black bar) pathways were shown.

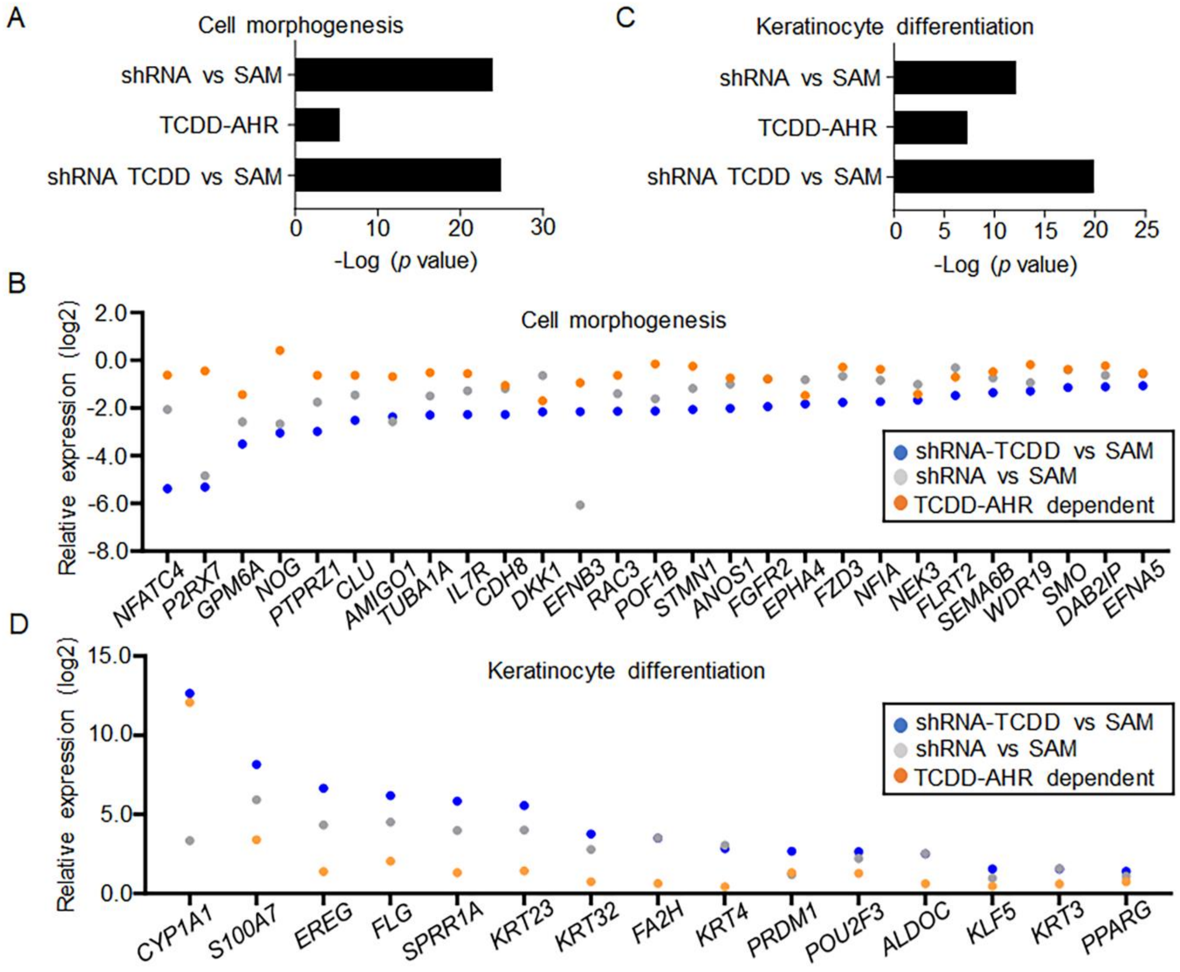

Figure S5. TCDD plus MAP3K1 deficiency affects cell morphogenesis and keratinocyte differentiation. Pathway analyses of the transcriptome data identify (**A**) cell morphogenesis as a function among genes down-regulated and (**C**) keratinocyte differentiation as a function among genes up-regulated by MAP3K1 (shRNA vs SAM), TCDD-AHR, and TCDD plus MAP3K1 deficiency (shRNA-TCDD vs SAM). The specific genes and their relative expression in (**B**) cell morphogenesis down-regulated by TCDD and/or MAP3K1 deficiency, and (**D**) keratinocyte differentiation up-regulated by TCDD and/or MAP3K1 deficiency.

Supplementary Tables

Table S1 *Map3k1* sgRNA sequences

sgRNA	Sequences 5'-3'
U6-g5 F	taa gca gaa gac atc acc gtg cgg ccg gga cta cc
U6-g5 R	taa gca gaa gac atg acg aaa aaa agc acc gac tcg g
U6-g12 F	taa gca gaa gac atc gtc ttt ttt tcg tgg ctg agc c
U6-g12 R	taa gca gaa gac atc ctg ctc ccg gag aaa ggg ta
U6-g13 F	taa gca gaa gac atc agg gag ggc cta ttt ccc atg att
U6-g13 R	taa gca gaa gac atc gcc aaa aaa agc acc gac tc
U6-g14 F	taa gca gaa gac atg gcg ttt ttt tcg tgg ctg agc c
U6-g14 R	taa gca gaa gac atc aac ctc ccg gag aaa ggg ta
U6-g15 F	taa gca gaa gac atg ttg gag ggc cta ttt ccc atg a
U6-g15 R	taa gca gaa gac att aaa acc gtc gtc ggg att ccc

Table S2 chemicals and reagents

Name	Company	Cat. No
Paraformaldehyde (96%)	Alfa Aesar	A11313.36
Mounting Medium Xylene	Fisher Health Care, Pittsburgh	245-691
CH-223191 (AHR antagonist)	Millipore Sigma	C8124
AG1478 (EGFR inhibitor)	Tocris Bioscience	1276
SP600125 (JNK inhibitor)	Calbiochem	420119
PD98059 (ERK inhibitor)	Cell Signaling Technologies	9900L
Fetal Bovine Serum (FBS)	R&D System	S11150
L-glutamine	Corning	25-005-Cl
Non-essential amino acid	Gibco	11140-050
Sodium pyruvate	Gibco	11360-070
Penicillin-Streptomycin	Cytiva	SV30010
Keratinocyte-Serum Free Medium (-) calcium chloride (KSFM-Ca)	Gibco	10725-018
Dulbecco's Modification of Eagle's Medium (DMEM)	Corning	10-017-CV
2,3,7,8-Tetrachlorodibenzo-p-dioxin (TCDD)	AccuStandard	D-404N
TrypLE™ Express	Gibco	12604-021
Sphingosine-1-phosphate (S1P)	Cayman chemical	26993-30-6
Hoechst 33342	Sigma	B2261
Dimethyl sulfoxide (DMSO)	Sigma	D2650
Puromycin	VWR	97064-280
Blasticidin	Thermo Fisher Scientific	R21001
Hygromycin	Calbiochem	400051
Sodium vanadate	Fisher Scientific	S454-50

Table S3 Antibodies

Target protein	Host	Company	Dilution ratio	Cat. No
CYP1A1	Rabbit	Absolute	1:100	Ab04076-23.0
MAP3K1	Rabbit	Made in house	1:500	
Phosphor-JNK	Rabbit	Cell Signaling Technologies	1:1000	4668S
Phosphor-ERK	Rabbit	Cell Signaling Technologies	1:1000	9101S
Phosphor-EGFR	Rabbit	Cell Signaling Technologies	1:1000	4407S
ERK	Rabbit	Cell Signaling Technologies	1:1000	9102
EGFR	Rabbit	LifeSpan BioSciences, Inc	1:1000	C117640
JNK	Rabbit	Invitrogen	1:1000	MA5-15183
E-cadherin	Mouse	BD Biosciences	1:100	610182
Par6	Mouse	Santa Cruz Biotechnology	1:100	sc-365323
Acetyl-tubulin	Mouse	Sigma	1:100	T7451
Keratin 1	Rabbit	BioLegend	1:100	905602
Anti-mouse IgG	Goat	Invitrogen	1:300	A11004
Anti-rabbit IgG	Goat	Invitrogen	1:300	A11034
Anti-mouse HRP	Goat	PIERCE	1:2000	1858413
Anti-rabbit HRP	Goat	PIERCE	1:2000	1858415

Table S4. Primers for RT-PCR

Gene	Forward 5'-3'	Reverse 5'-3'
GAPDH	tcg gag tca acg gat ttg gtc gta	tga tga caa gct tcc cgt tct cag
CYP1A1	ttt gag aag ggc cac atc cg	gat agc agt tgt gac tgt gtc a
IER3	tca cca tgt gtc act ctc gc	aga agc ctt ttg gct ggg tt
EREG	atc aca gtc gtc ggt tcc ac	agg cac act gtt atc cct gc
EGR1	tgg gtt tga tga gct ggg ac	ccc cga cta cct gtt tcc ac
FOSL1	cct cag ctc atc gca aga gt	aca ttg gct agg gtg gca tc
DUSP4	gag cca ggt tgt ctt gtg ga	caa aaa gcc tgg aag ggt gc
ETV5	agg ggc aga aaa cca cca aa	gtc ccg ttt tgc ggg tac ta
MAP3K1	cat cag gtc gca cag tga aat	tca ggg cta tat ggt gag aag c
FLG	gac acc ccg gat cct ctc acc	agc tgc cat gtc tcc aaa cta aac
KRT1	ttc atc gac aag gtg cgc ttc cta	tgg tca cga act cat tct ctg cgt
KRT10	tga atg tgg aaa tga acg ctg ccc	agt agc gac ctt ctg ttt ctg cca
ALPP	agg ctt gcc cca aat ctc aa	gac ctt tgg ctc tcg acc ag
PTPRE	gac ttc gga gtg cct ttt acc	gag ggc gct gat tac gga tt
ALPPL2	ggc atc atc cca gtt gag gag	gca cat gct tgt cta cac tgt at
PFKFB4	caa cat cgt gca agt gaa act g	gac tcg tag gag ttc tca tag ca
